# The Once and Future Fish: Assessing a Millennium of Atlantic Herring Exploitation Through Mixed‐Stock Analysis and Ancient DNA

**DOI:** 10.1111/gcb.70010

**Published:** 2024-12-26

**Authors:** Lane M. Atmore, Inge van der Jagt, Aurélie Boilard, Simone Häberle, Rachel Blevis, Katrien Dierickx, Liz M. Quinlan, David C. Orton, Anne Karin Hufthammer, James H. Barrett, Bastiaan Star

**Affiliations:** ^1^ Centre for Ecological and Evolutionary Synthesis, Institute of Biosciences University of Oslo Oslo Norway; ^2^ Department of Anthropology University of British Columbia Vancouver British Columbia Canada; ^3^ Cultural Heritage Agency of the Netherlands Amersfoort The Netherlands; ^4^ Integrative Prehistory and Archaeological Science, Department of Environmental Sciences Basel University Basel Switzerland; ^5^ Department of Archaeology University of Cambridge Cambridge UK; ^6^ Department of Archaeology and Cultural History, NTNU University Museum Norwegian University of Science and Technology (NTNU) Trondheim Norway; ^7^ Department of Archaeology University of York York UK; ^8^ Department of Archaeology University of Bergen Bergen Norway

**Keywords:** ancient DNA, ecology, fisheries, mixed‐stock analysis, sustainable management

## Abstract

Small pelagic fish support profitable fisheries and are important for food security around the world. Yet, their sustainable management can be hindered by the indiscriminate impacts of simultaneous exploitation of fish from multiple distinct biological populations over extended periods of time. The quantification of such impacts is greatly facilitated by recently developed molecular tools—including diagnostic single nucleotide polymorphism (SNP) panels for mixed‐stock analysis (MSA)—that can accurately detect the population identity of individual fish. However, the biological relevance of such tools over longer periods of time remains unknown. Here, we demonstrate that diagnostic SNP panels designed for contemporary MSA in Atlantic herring have a millennium‐long biological relevance and applicability. We assign the population identity of ancient Atlantic herring specimens—obtained through famously profitable historic fisheries—up to 1300 years old from eight archaeological sites across Europe. Analyzing contemporary and ancient whole‐genome data, we obtain evidence for the long‐term mixed‐stock exploitation of Atlantic herring. Despite such mixed‐stock exploitation, we exclusively identify autumn‐spawning herring amongst these archaeological remains, indicative of a specific biological availability or cultural preference for certain herring ecotypes in the past. Moreover, our results show that herring demographic patterns were relatively stable until the dramatic disruptions and stock collapses during the 20th century. We find small but significant reductions in genetic diversity over time, indicating long‐term evolutionary consequences from 20th‐century stock declines. The long‐term applicability of diagnostic SNP panels underscores their biological relevance and cost‐effective application for the genetic monitoring of herring stocks and highlights the utility of ancient DNA to obtain insights in herring ecology and population dynamics.

## Graphical abstract

Ancient DNA was extracted from archaeological herring bones and used to determine region of origin for ancient samples. We find that SNP panels designed for contemporary mixed‐stock analysis identify loci that are largely unchanged over a millennium, suggesting these panels are a cost‐effective method for fisheries management. Finally, we discover that genetic diversity in the Atlantic herring has been lost over time, indicating there has been a genetic impact from overfishing in the 20th century.

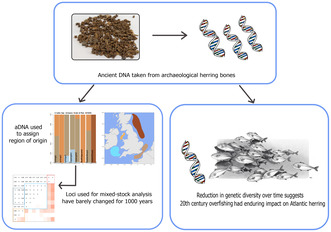

## 1 Introduction

Small pelagic fish made up six of the 10 largest fisheries in the world in 2021, comprising a significant portion of global landings (FAO 2024). These fish play important roles in local economies, and as crucial elements in energy transfer between trophic levels in marine ecosystems around the world (Pikitch et al. 2014). In recent years, small pelagic fish have been touted as the more sustainable choice for consumers in the western world, as they are argued to have a lower carbon footprint than popular species such as salmon and tuna (Koehn et al. 2022) (although see Thurstan and Roberts 2014 for a critique of this suggestion). These species are also increasingly being used for non‐direct consumption such as in fish meal for aquaculture and fertilizer, driving demand for increased yield, and creating issues of food security (Isaacs 2016). Yet, small pelagic fish face intense challenges due to both overfishing and climate change, thus requiring nuanced approaches for sustainable management (Froese, Papaioannou, and Scotti 2022; Niiranen et al. 2013).

Small pelagic fish such as the Atlantic herring often comprise biologically distinct spawning populations that occur intermingled during migration and feeding phases, thus modern fishing practices can result in mixed‐stock exploitation (Bekkevold et al. 2023). Such indiscriminate mixed‐stock exploitation has complicated efforts to introduce sustainable fishing management (Moore, Connors, and Hodgson 2021), as expert policy advice recommends management based on biological populations rather than a mixed stock to improve sustainable exploitation and reduce portfolio effects (Bartolino et al. 2023). Novel molecular tools have been developed in an effort to address the issue of mixed‐stock exploitation, but they are resource‐ and time‐intensive to create and their long‐term viability is yet to be determined (Bekkevold et al. 2023; Farrell et al. 2022a). Sustainable management is further complicated by the long history of exploitation experienced by many small pelagic fish species, which have often exhibited dramatic population fluctuations due to climate change and overexploitation over the past millennium (Atmore et al. 2022a). We here investigate the evolutionary impacts of long‐term exploitation and the viability of diagnostic SNP panels for mixed‐stock analysis (MSA) over long timescales using the Atlantic herring ( *Clupea harengus* ), which has a long history of exploitation in northern Europe.

### 1.1 A Long History of Herring Exploitation

The Atlantic herring is a small pelagic fish that has been a crucial source of food and economic activity in the nations surrounding the North Sea for well over a thousand years (Barrett 2016; Hoffmann 2001; Kowaleski 2016). Historical fishing operations in the North Sea likely targeted the North Sea autumn‐spawning herring, which, until the 1970s, supported one of the largest herring fisheries in the world (Dickey‐Collas et al. 2010; Poulsen 2008). In England, fishers would migrate down the coast of the North Sea following spawning aggregations; herring was so abundant c. 1000 CE that it was called “herringsilver” and used as payment in tithes and taxes (Kowaleski 2016). The marked increase in herring remains in archaeological sites in England following 1000 CE, alongside the appearance of large quantities of other marine fish species, has led archaeologists to suggest—somewhat tongue‐in‐cheek—that this date represents the “Fish Event Horizon” (FEH); a point at which marine populations were exploited at scale for the first time in this region (Barrett, Locker, and Roberts 2004). Perhaps the most famous and most extensively studied herring fishery in the North Sea was that of the Dutch Republic in the 16th–18th centuries, peaking between the late 1500s and 1600s (Holm et al. 2022; Poulsen 2008). Fishing expansion in Northern Europe during this time was so extensive that it represents a second phase of commercialization following the “FEH” in the 11th century (Barrett, Locker, and Roberts 2004; Kowaleski 2003). Herring was then a profitable commodity, and the Dutch Republic controlled up to 80% of the market, resulting in an influx of wealth that supported the “Dutch Golden Age” of scientific and political experimentation as well as global exploration and colonization (Poulsen 2008). Herring was so important to the Netherlands it was called “Gratia Dei” (“gift from god”), a “golden mountain,” and the “triumph of Holland” (Coenen 1577).

While historians modeling catch records initially concluded that historical herring industries could not have impacted herring stock size (Poulsen 2008) recent research suggests landings across Europe were far larger than previously understood (Holm et al. 2022). By the 20th century, herring throughout the eastern Atlantic were heavily exploited with little regulation, resulting in stock collapses (Dickey‐Collas et al. 2010; Schmidt et al. 2009; Toresen and Jakobsson 2002). Despite warnings from the International Council for the Exploration of the Sea (ICES) Herring Assessment Working Group (HAWG), management bodies failed to reduce total allowable catch (TAC) in the North Sea until the population had been nearly entirely wiped out. By this time, North Sea Autumn Spawners (NSAS) spawning‐stock biomass (SSB) is estimated to have declined by 97%, from 5 million tonnes in 1940 to 50,000 t in 1970 (Dickey‐Collas et al. 2010). By 2010, SSB had recovered to 1.5 million tonnes (Dickey‐Collas 2016; Dickey‐Collas et al. 2010), but this success was shortly followed by recruitment failures again in recent years (Bartolino et al. 2023). The North Sea herring fishery currently accounts for 21% of the total economic value of the EU pelagic fisheries sector, thus ongoing recruitment failures will have widespread impacts on the EU economy as well as the North Sea ecosystem (Rybicki et al. 2020). In recent years, ICES has again recommended reductions in TAC for the NSAS, as recruitment continues to falter (Bartolino et al. 2023). Landings for fisheries in the North, Celtic, and Irish Seas (the regions encompassed by sampling in this study) are consistently higher than the recommended TAC, and stocks are assessed as in varying degrees of health (evaluated based on stock size estimates) (Bartolino et al. 2023).

A key aim in determining sustainable management policies is establishing what constitutes healthy ecosystem thresholds (Spake et al. 2022). Understanding ecosystem thresholds necessitates acknowledging that exploitation may have occurred for extended periods of time, thus efforts must take historical exploitation levels into account rather than relying on landing records that typically begin in the 20th century—a time at which marine resources were likely already heavily depleted (Roberts 2007). Efforts to quantify historical impact have revealed a long history of marine exploitation through archaeological and historical research (Barrett 2016; Holm et al. 2022). Nonetheless, assessing the impact of historical fisheries of small pelagic fish hinges on correctly identifying the biological populations that were targeted in the past, and for many archaeological fish bone assemblages origin cannot be discerned through morphology. The application of ancient DNA provides fine‐scaled resolution in determining population of origin for archaeological remains and allows assessment of shifting exploitation patterns over time, both through identifying ancient trade routes and by determining whether a specimen was locally fished (Atmore et al. 2022a; Der Sarkissian et al. 2020; Ferrari et al. 2022; Guimaraes et al. 2020; Star et al. 2017). Identifying population of origin will further allow for direct biological comparison of samples across time, allowing the assessment of shifts in genomic diversity, demography, and population continuity (Atmore et al. 2022a). Thus, aDNA is a crucial tool for addressing ongoing questions in herring demography and evolution, such as the impact of historical industrial‐scale fisheries and how herring respond to dramatic shifts in climate (e.g., Medieval Climate Anomaly, Little Ice Age), thereby facilitating understanding of the impact of contemporary and future anthropogenic and climatic changes.

### 1.2 MSA of Atlantic Herring

To improve the management of Atlantic herring, novel genetic resources have been developed for MSA. For example, through the creation of diagnostic SNP panels that can be used to assign biological population of origin to individual fish that are exploited together and with the aim that these can at some point be applied to a revision of current ICES subdivisions and recommendations (Bekkevold et al. 2023; Farrell et al. 2022a). These tools have high statistical power to identify the biological population of a specific individual, even in those cases with low overall population differentiation. For instance, based on such tools the English Channel (Downs) and NSAS populations were divided into separate ICES management units in 2022 (Bartolino et al. 2023; Bekkevold et al. 2015, 2016; Farrell et al. 2022a). However, many of these tools are still under development and it remains unknown how well these tools perform over long periods of time and if diagnostic SNP panels used for MSA will need to be reassessed frequently (Bekkevold et al. 2023). Understanding how well these diagnostic SNP panels work over time will facilitate their development and implementation by providing information on their cost‐effectiveness and the underlying evolutionary processes that provide their statistical power and utility. For example, loci associated with local adaptation that are driving population structure may have changed over time as the species experienced environmental change, providing crucial information for predicting how populations may respond to future climate change.

In recent years, the application of assignment tools to ancient specimens has demonstrated population structure continuity on the level of metapopulation for several marine fish, including Atlantic cod (Martínez‐García et al. 2022) and Atlantic herring (Atmore et al. 2022a). Previous ancient DNA studies for Atlantic herring have focused on assigning population to relatively broad metapopulations based on spawning season and local adaptation (e.g., salinity levels). We here use ancient DNA and whole‐genome analysis to assess spatiotemporal changes in mixed‐stock exploitation of herring in the North Sea, English Channel, and Celtic and Irish Seas by applying genetic MSA panels to archaeological herring remains spanning the 8th–16th centuries. We discover that modern diagnostic SNP panels designed for MSA in sustainable management regimes have deep‐time relevance by identifying the biological origin of archaeological herring remains up to 1300 years old. We further demonstrate changes in genome‐wide herring population structure and genetic diversity over time consistent with population decline. Finally, we model effective population size over time to determine the timing of these changes.

## 2 Materials and Methods

### 2.1 Sampling

Modern data were accessed from publicly available datasets (Atmore et al. 2022b; Han et al. 2020b). The modern dataset comprises 68 individuals from all major herring populations in the Baltic and eastern Atlantic. Archaeological herring material comprising 32 individuals was sourced from 8 sites across Europe spanning the 8th–16th centuries (see Data S1 for full archaeological context data) (Atmore et al. 2024a).

### 2.2 DNA Extraction and Sequencing

Archaeological samples were processed following established ancient DNA laboratory protocols to minimize the risk of contamination (Gilbert et al. 2005; Llamas et al. 2017). DNA extractions took place at the dedicated ancient DNA laboratory at the University of Oslo. Libraries were prepared with both double‐stranded (Meyer and Kircher 2010) and single‐stranded (Kapp, Green, and Shapiro 2021) methods. All libraries were purified using AMPure magnetic bead purification and assessed for quality with the Agilent DNF‐474 High‐Sensitivity kit on an Agilent Fragment Analyzer 5200. Libraries with sufficient quality and no indication of contamination were sequenced on an Illumina HiSeq 4000 and/or NovaSeq 6000 at the Norwegian Sequencing Centre. 32 archaeological sequences were generated (Atmore et al. 2024b). Full metadata for the modern samples is included in Data S2 (Atmore et al. 2024a). All sequences were aligned to the herring reference genome Ch_v2.0.2 (Pettersson et al. 2019) using the PALEOMIX (Schubert et al. 2014) v2 pipeline and bwa‐mem (Li and Durbin 2010). mapDamage2.0 (Jónsson et al. 2013) plots were generated to assess post‐mortem deamination patterns and validate the ancient samples (Figure S1). Archaeological samples yielded 0.0001–23X coverage. Coverage for modern sequences ranged from 7 to 55X.

### 2.3 Dataset Curation

Three datasets were created: modern data, low‐coverage ancient and modern data, high‐coverage ancient and modern data. The modern dataset was used to provide training data and a reference database for assignment tests using BAMscorer. All ancient sequences were used for population assignment tests (see below). Sequences with missingness < 30% and coverage > 2× were included in a low‐coverage dataset in combination with the modern sequences for analysis using angsd, a program designed for low‐coverage sequence data (Korneliussen, Albrechtsen, and Nielsen 2014) (16 ancient and 68 modern samples). High‐coverage ancient sequences and modern data were combined for SNP calling using GATK (McKenna et al. 2010) (8 ancient and 68 modern samples). To confirm that all archaeological specimens were Atlantic herring, the mitogenomes were aligned to the reference using the Paleomix pipeline (see above). SNPs were called with GATK for all ancient and modern specimens, filtered for quality, then a maximum‐likelihood phylogenetic tree was built with IQ‐TREE v1.6.12 using default settings (Nguyen et al. 2015) (Figure S2). A Pacific herring ( *Clupea pallasii* ) individual was used as the outgroup (Han et al. 2020b). All mitogenome sequences were assessed for intraspecific cross‐sample contamination by examining the rates of heterozygosity with VCFtools (Danecek et al. 2011) after calling loci as pseudo‐diploid; as a haploid sequence, non‐contaminated individuals should have a heterozygosity rate of 0 using this method. Any individuals with heterozygosity levels above 0 were removed from the dataset.

### 2.4 SNP Calling

Filtering settings were determined after examining characteristics of the raw VCF files of the final high‐coverage and modern datasets, including minor allele frequency, individual depth, locus depth, and locus quality with bcftools (Danecek et al. 2021), VCFtools, and R (R Core Team 2021). High‐coverage datasets were filtered as follows: BCFtools: FS< 60.0 && SOR< 4 && MQ> 30.0 && QD> 2.0 && INFO/DP< 415140 –SnpGap 10; VCFtools: ‐‐minGQ 15 ‐‐minDP 3 ‐‐remove‐indels ‐‐maf 0.01 ‐‐max‐missing 0.9; and GATK: –restrict‐alleles‐to BIALLELIC. The modern dataset contained 4,317,675 SNPs. For smartPCA and GONE demographic analysis, no maf filtering was applied, as removing minor alleles can artificially reduce signals of population structuring and demographic change (Linck and Battey 2019; Santiago et al. 2020). The non‐maf‐filtered dataset contained ~10 million SNPs. An additional dataset was created by removing previously identified inversion sites (Han et al. 2020a) and LD‐pruning with PLINKv1.9 (Purcell et al. 2007) (‐‐exclude range inversions.list ‐‐indep‐pairwise 100 10 0.5), resulting in 2,846,566 SNPs. The high‐coverage dataset (including 8 ancient individuals) contained 7,334,073 variants after filtering, with the LD‐pruned dataset containing 5,673,945 variants. For runs of homozygosity (ROH), the high‐coverage dataset was further reduced to include individuals with < 5% missingness, resulting in only two ancient individuals remaining (both from the same context of the 9th century site Lyminge).

### 2.5 Population Structure and MSA (Contemporary)

Population structure was investigated for the non‐maf‐filtered modern dataset using smartPCA (Patterson, Price, and Reich 2006). Hudson's pairwise *F*_ST_ (Hudson, Slatkin, and Maddison 1992) and kinship coefficients were calculated using popkin (StoreyLab/Popkin 2017/2023), both with the full dataset and with a subset of SNPs putatively under selection determined through PCAdapt (Privé et al. 2020) (Figures S3 and S4). To further fine‐tune population assignment, diagnostic SNPS for MSA (Bekkevold et al. 2023; Farrell et al. 2022b) were extracted from the modern and ancient samples identified as belonging to the waters surrounding Britain and Ireland and the North Sea (BINSA). 82 loci in total were called from contemporary samples using angsd (‐sites binsa_sites.list ‐uniqueOnly 1 ‐remove_bads 1 ‐only_proper_pairs 1 ‐trim 0 ‐C 50 ‐baq 1 ‐minMapQ 30 ‐minQ 20 ‐noTrans 1 ‐doCounts 1 ‐GL 1 ‐dobcf 1) (Atmore et al. 2024a). Individuals with > 20% missing loci were removed. Population substructure assessment was conducted in R using DAPC from the adegenet package (Jombart 2008). DAPC was conducted first on the modern data only to confirm the ability of the diagnostic SNPs to assign population as reported in previous studies. The identified subpopulations were used to inform subsequent analysis.

### 2.6 Population Assignment of Archaeological Specimens

**FIGURE 1:**
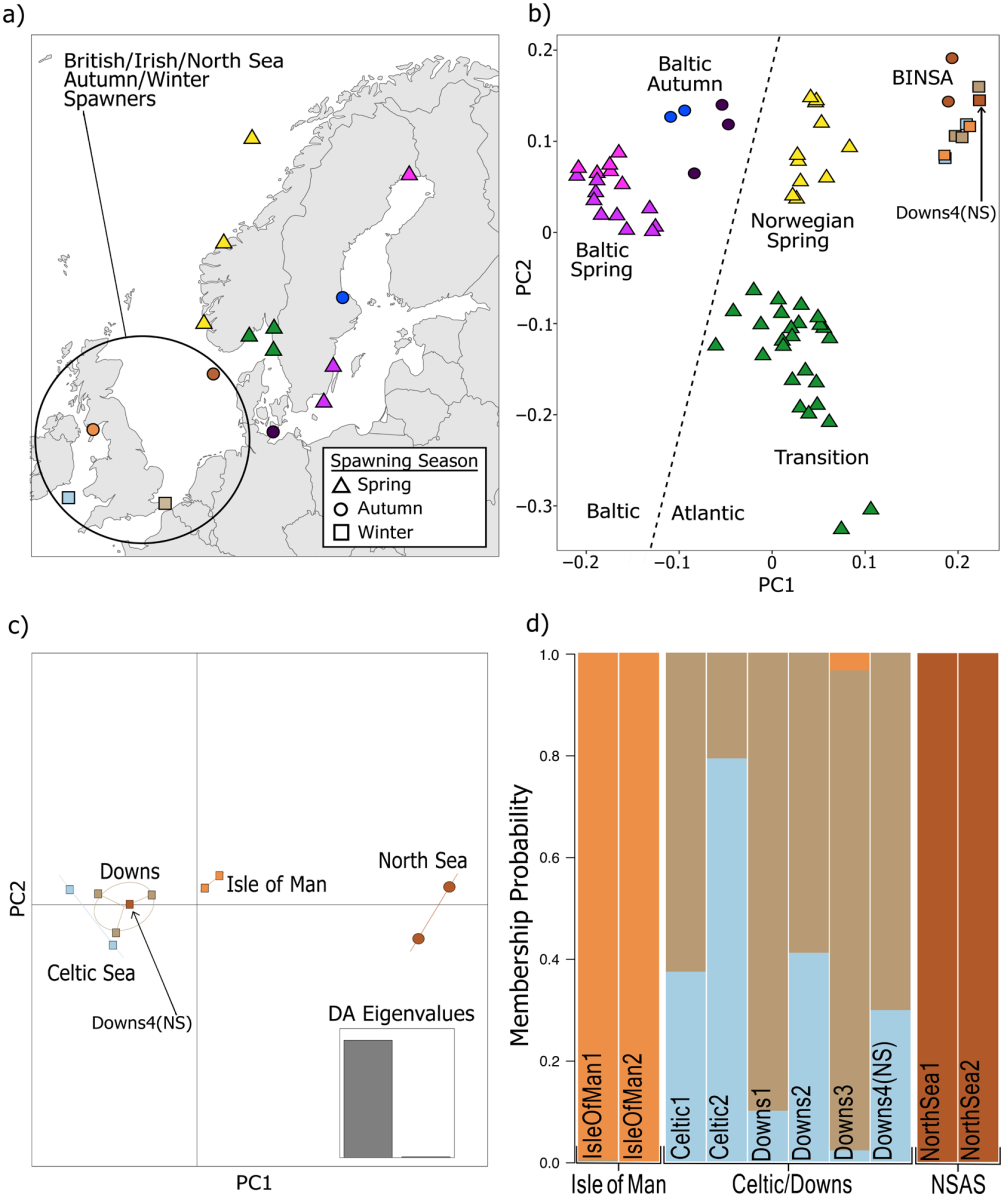
Population structure in modern Atlantic herring is based on local adaptation. (a) Sampling locations for modern herring samples. Shapes correspond to spawning season; (b) herring are separated into metapopulations corresponding to adaptive phenotypes associated with spawning phenotype and local salinity levels. Samples are colored by sampling location (panel a) and shapes correspond to spawning season. PCA constructed with 10 million SNPs (non‐maf filtered) using smartPCA; (c) mixed‐stock analysis with the BINSA metapopulation shows substructuring between the North Sea, Isle of Man, and Celtic/Downs populations. This panel shows the results of DAPC analysis using two PCs; and (d) three subpopulations are indicated by DAPC analysis: North Sea, Isle of Man, and an admixed population between Celtic Sea and Downs based on 82 SNPs diagnostic SNPs designated for mixed‐stock analysis. Four populations were used as input priors: Celtic Sea, Downs, Isle of Man, and North Sea (NSAS). DAPC suggests there are three groups: Downs4(NS) was identified as an admixed Celtic Sea/Downs individual, not a North Sea individual as listed in public metadata. Samples are grouped based on genetic identity (labeled on *x*‐axis). Labels within bars refer to individual sample IDs. Map lines delineate study areas and do not necessarily depict accepted national boundaries.

Population assignment tests were conducted on the archaeological samples using BAMscorer v1.6.1 (Ferrari et al. 2022). Following the methods detailed in Atmore et al. (2022a), sensitivity analysis was performed for three levels of assignment test based on inversion type and ecological adaptation: salinity adaptation, spawning season, and chromosome 12 inversion haplotype. Spawning season could be determined with as few as 50,000 reads; the chromosome 12 inversion could be assigned with as few as 5000 reads; and salinity scores could be determined for samples with at least 60,000 reads. All samples were assigned to one of the following populations: British/Irish/NSAS (BINSA); Norwegian Spring spawners (NSS); Transition zone spring spawners (Transition); Western Baltic Autumn Spawners (WBAS); Central Baltic Autumn Spawners (CBAS); Central Baltic Spring Spawners (CBSS); and Gulf Spring Spawners (GSS) (see Figure 1). All archaeological samples identified as BINSA and with sufficient sequence quality were further investigated with DAPC using the diagnostic SNP panels for MSA. 82 loci in total were called from the ancient high‐coverage using angsd (‐sites binsa_sites.list ‐uniqueOnly 1 ‐remove_bads 1 ‐only_proper_pairs 1 ‐trim 0 ‐C 50 ‐baq 1 ‐minMapQ 30 ‐minQ 20 ‐noTrans 1 ‐doCounts 1 ‐GL 1 ‐dobcf 1). Transitions were removed (‐noTrans 1) in order to minimize the potential impact of deamination damage. Individuals with > 20% missingness were removed from analysis. To assess the temporal robustness of these diagnostic loci, divergence between the ancient and modern samples were assessed using Jost's *D* (Jost et al. 2018) as a measure of genetic divergence among demes.

### 2.7 Genetic Diversity Changes Across Time

Heterozygosity was measured using angsd on an individual basis for the dataset including ancient samples. To calculate heterozygosity per individual, the individual folded site‐frequency spectrum (SFS) was generated using the reference genome as ancestral and standard quality filters (‐C 50 ‐minMapQ 20 ‐minQ 20 ‐rmTrans 1). Individual folded SFS were generated with angsd (filters: ‐uniqueOnly 1 ‐remove_bads 1 ‐only_proper_pairs 1 ‐trim 0 ‐C 50 ‐minMapQ 30 ‐minQ 20 ‐setMinDepth 5 ‐setMaxDepth 60 ‐rmTrans 1) by chromosome. Given the potential impact of deamination in the ancient samples due to post‐mortem damage, transitions were removed from the dataset (flag –rmTrans 1 in angsd). Chromosome‐level SFS were then used to calculate individual heterozygosity ranges across the entire genome with realSFS, which includes invariant sites. ROH were further explored for all samples with < 5% missingness using loci called with angsd (using the above quality filters, including removing transitions). Our previous analysis suggests that ROH estimates are strongly biased by sampling size when overall sampling sizes are small (Atmore et al. 2022a), therefore all populations were randomly downsampled to two samples each. ROH were assessed using PLINK v1.9 for all samples with < 5% missingness with the following commands: ‐‐chr‐set 26 ‐‐double‐id ‐‐homozyg‐snp 50 ‐‐homozyg‐kb 90 ‐‐homozyg‐density 50 ‐‐homozyg‐gap 1000 ‐‐homozyg‐window‐snp 50 ‐‐homozyg‐window‐het 3 ‐‐homozyg‐window‐missing 10 ‐‐homozyg‐window‐threshold 0.05. To account for the difference in sample size between populations, all populations were sampled to 2 individuals each. Age of coalescent event per ROH was calculated using the following formula: *L* = 100/2 g cM (Kirin et al. 2010) (Figure S5).

Treemix (Pickrell and Pritchard 2012) was run on the LD‐pruned whole‐genome dataset with a Pacific herring individual as outlier (missingness set to 0). Treemix showed no divergence between the BINSA subpopulations with the full genome dataset (Figure S6), therefore the diagnostic SNPs from the MSA were extracted from each sample and Treemix was rerun on the smaller dataset (Figure S7). Treemix was bootstrapped through 10 iterations per migration edge (0–5) and then an optimal migration edge was selected using OptM (Fitak 2021) (Figure S8). The optimal migration edge was then used as a setting for bootstrapping and visualization with BITE (Gaboriau et al. 2020).

### 2.8 Demographic Modeling

The non‐maf‐filtered modern dataset was used to estimate effective population size (*N*_e_) throughout time with GONE (Santiago et al. 2020). The BINSA population was assessed both as a homogenous population and by geographic sampling location to determine the possibility of cryptic population substructure and to control for the difference in sampling time between the oldest and newest specimens (~50 years = up to 15 generations). GONE results were used to confirm MSA with DAPC by assessing the presence or absence of artifacts known to be produced by admixture (Novo et al. 2023) (Figures S9 and S10). Known inversions (Han et al. 2020a) were removed from the dataset prior to running GONE. GONE internally bootstraps *N*_e_ estimation through 40 iterations for each generation using a random subsampling of 50,000 SNPs from each chromosome per iteration. The program provides a geometric mean population size per gen. To assess reliability of the program given known sensitivity to low sample sizes (Santiago et al. 2020), each population from the modern dataset was run through GONE 30 times, with the median population size estimate per gen taken as the consensus effective population size at a given time depth. Default parameters were used other than including the recombination rate for the Atlantic herring (2.54 cM/Mb) (Han et al. 2020a; Pettersson et al. 2019). Time depth was estimated using 3–5 years as a minimum generation time for Atlantic herring based on average generation time per subpopulation computed from Feng et al. (2017). Demographic trend lines were plotted against sea‐surface temperature reconstructions from Mann et al. (2009) (extracted data can be accessed via Atmore et al. 2024a).

## 3 Results

### 3.1 Population Structure and MSA (Contemporary)

Atlantic herring are structured into metapopulations that correspond to adaptive phenotypes associated with local salinity levels and spawning season, as shown by principle components analysis (PCA) analysis of high‐coverage whole‐genome data of 68 specimens from across Europe (Figure 1) (Atmore et al. 2022a; Han et al. 2020a). Atlantic and Baltic herring cluster separately with autumn/winter spawners segregating from spring spawners within these two clusters (Figure 1a). An additional cluster lying outside of this grouping comprises Transition Zone populations, which spawn in the spring and live in the Kattegat and Skagerrak area between the Baltic and the North Sea. Salinity levels in this region are similar to the Atlantic, thus they cluster closer to the Atlantic populations on the PCA. The herring stocks under consideration in this study—British, Irish, and North Sea Autumn/Winter spawners (BINSA)—clustered together in one metapopulation on the PCA (Figure 1a). This metapopulation can be split into three subpopulations: Isle of Man, Celtic Sea/Downs, and North Sea (NSAS) based on MSA using 82 SNPs that discriminate between these herring populations in the North Atlantic (Bekkevold et al. 2023; Farrell et al. 2022a). MSA suggests the North Sea subpopulation is more distinct from individuals sampled at the other three locations (Celtic Sea, Downs, and Isle Of Man) and Downs and Celtic Sea populations are unable to be distinguished based on this SNP panel (Figure 1c,d), which concurs with previous studies (Farrell et al. 2022a).

One of the modern individuals listed as sampled within the NSAS population showed high similarity to the Downs/Celtic Sea population (Figure 1b; Figure S11). This sample was not collected in a region where NSAS typically spawn, but in a region associated with the feeding migration (Daan et al. 1990; Han et al. 2020a). We therefore considered this a mixed‐stock sample and re‐classified this individual as genetically belonging to the “Downs” population in all subsequent analyses (Downs4(NS)). Herring population structure analyzed using discriminant analysis of principal components (DAPC) (Jombart 2008) shows that the optimum number of PCs to describe the modern data is 1 (Figure S12) and the estimated number of true populations yields *K* = 2, based on the DAPC *find.clusters* function. These results indicate that the most significant population structure is exhibited between the NSAS and all other BINSA subpopulations. Winter spawners south and west of the UK have a monophyletic relationship based on Treemix analysis of the diagnostic SNP panel (Figure S7). A single migration event from the common ancestor of the western BINSA populations to the North Sea was detected using OptM (Figures S7 and S8). Bootstrapping revealed lack of consensus on the placement of the edge, thus the consensus tree shows gene flow from the MRCA of Isle Of Man, Downs, and Celtic Sea to the contemporary North Sea population. No population differentiation between BINSA subpopulations was found using Treemix based on LD‐pruned whole‐genome data (Figure S6). This result corroborates that herring population structure is restricted to a few highly differentiated genomic regions.

### 3.2 Archaeological Specimen Assignment

**FIGURE 2:**
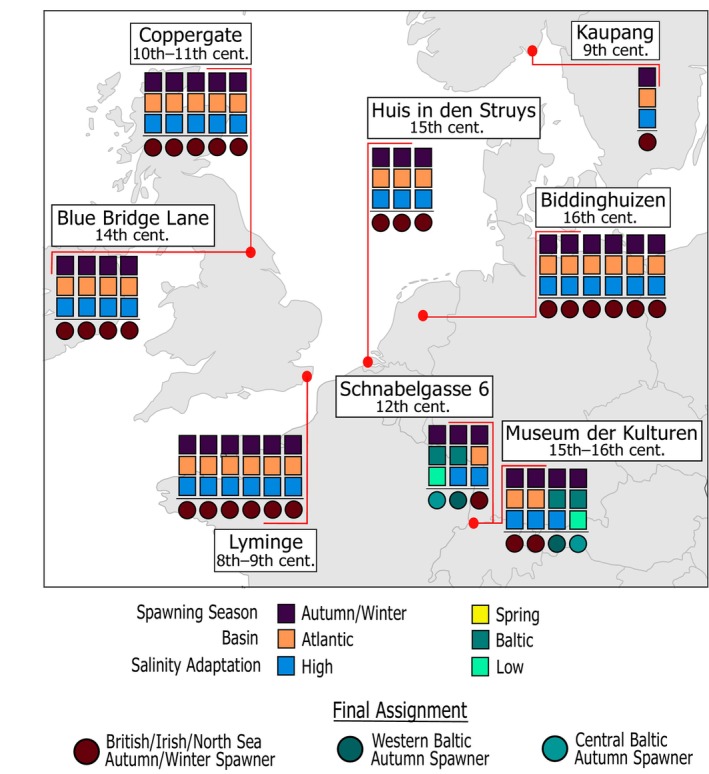
Metapopulation assignment for archaeological remains shows all archaeological herring are autumn spawners. Biological population assignment using BAMscorer for low‐and high‐coverage ancient herring remains. Samples are assigned to a final metapopulation through three hierarchical assignment tests. Each square represents the results of one assignment test: Spawning season (spring vs. autumn/winter), ocean basin (basin can be determined from the chromosome 12 inversion for autumn spawners), and salinity adaptation (high/low/mid). Circles represent the final BAMscorer assignment according to the combined assignment tests. Each column corresponds to one specimen. Two samples each from Schnabelgasse 6 and Museum der Kulturen originate from the Baltic, whereas all other samples are from the BINSA metapopulation. Map lines delineate study areas and do not necessarily depict accepted national boundaries.

**FIGURE 3:**
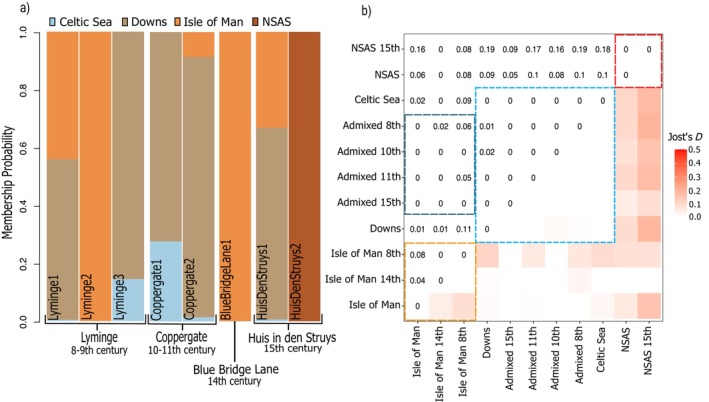
High‐coverage genomic analyses of ancient herring specimens show historic mixed‐stock exploitation and long‐term population substructure in the BINSA metapopulation. (a) Archaeological samples are assigned to each of the three subpopulations identified for the contemporary samples: Isle of Man, North Sea, and Celtic/Downs. The presence of herring from different spawning populations in Lyminge and Huis in den Struys is consistent with historic mixed‐stock exploitation. The ancient remains show a greater degree of genetic admixture between Downs and Isle of Man compared to modern specimens. Colors correspond to input population priors based on sampling locations for contemporary stocks; (b) persistent population substructure across time is shown through absolute divergence analysis based on subpopulations separated by time depth. Intrapopulation Jost's *D* throughout time largely shows values of 0 for Celtic/Downs and North Sea. Higher Jost's *D* is exhibited within IsleOfMan over time, whereas IsleOfMan samples show more similarity with ancient Celtic/Downs populations (dark blue hashed box), likely reflecting increased admixture with Celtic/Downs in the past. Labels refer to populations separated by time period to account for possible change over time. Hashed lines show the three major populations: IsleOfMan (orange), Celtic/Downs (blue), and NorthSea (red). “Admixed” refers to the ancient individuals that showed admixture between Isle of Man, Celtic Sea, and Downs in varying degrees.

**FIGURE 4:**
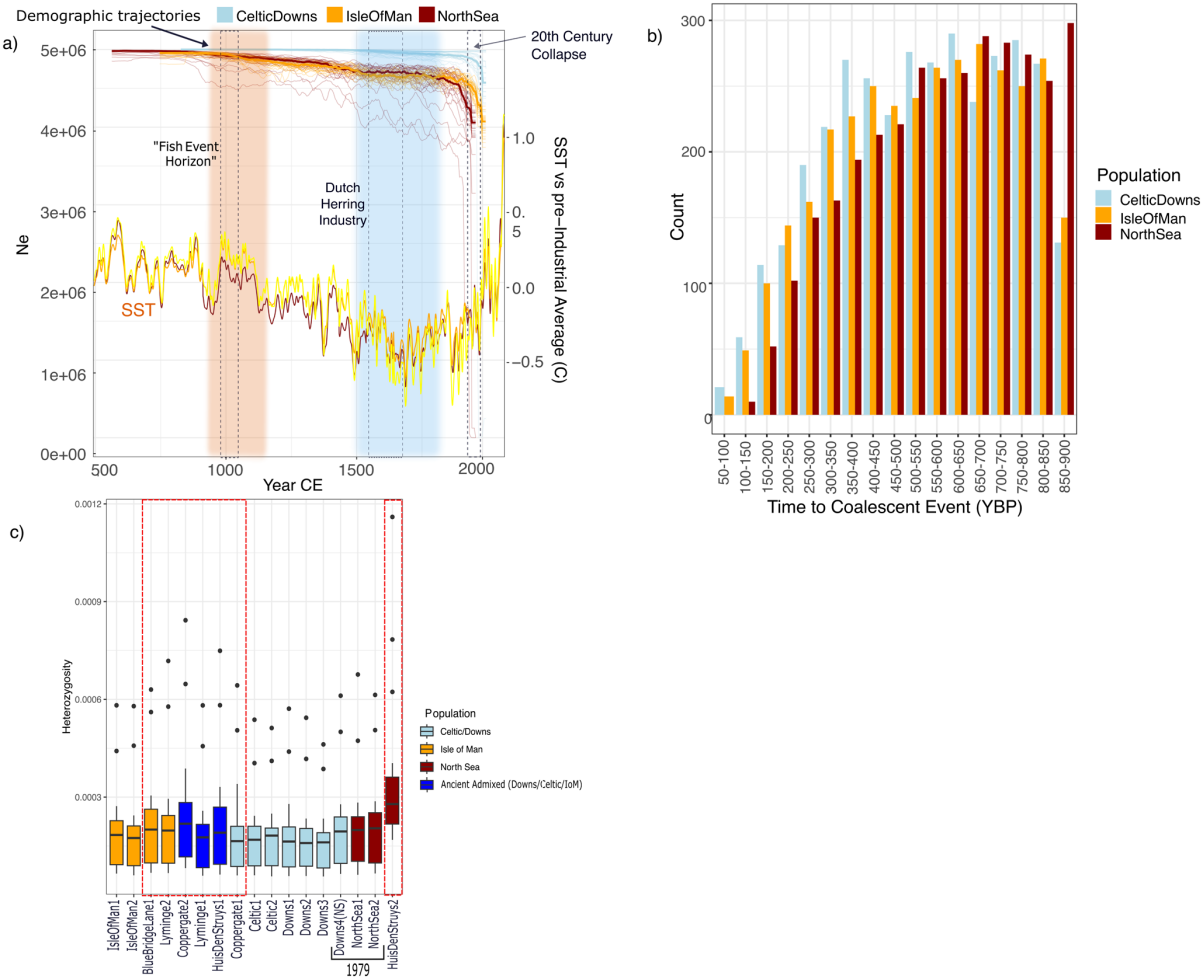
Genetic diversity and demographic change over time and space suggest recent population declines in exploited herring stocks. (a) *N*_e_ reconstructions showing recent dramatic declines, with population declines beginning ~1000 CE for NSAS and Isle of Man. *N*_e_ is estimated per population with GONE. Opaque lines represent median *N*_e_ estimates from 30 iterations of GONE, with each iteration represented by semi‐transparent lines. Year was calculated by scaling generational *N*_e_ estimates to generation times reported by Feng et al. (2017). Colored blocks represent the Medieval Climate Anomaly (orange) and Little Ice Age (blue). Hashed blocks represent historical events. Sea‐surface temperature (SST) reconstructions were adapted from three published indicators (Mann et al. 2009). (b) Number of ROHs associated with a coalescent event at a specific time period (calculated with the formula *T* = 100/2 × cM × Gen) per modern population. (c) Higher heterozygosity in ancient/historical samples (red hashed boxes) than 21st century samples. Heterozygosity was estimated per chromosome by individual using the site frequency spectrum. Samples are grouped according to site/basin of origin and boxplots are filled according to population assignment from MSA.

Archaeological specimens (*n* = 32) from eight different sites dated to the 8th–16th centuries were assigned to metapopulation by determining their adaptation to differing salinity levels (e.g., Baltic vs. Atlantic) and spawning season with BAMscorer v1.6. All ancient specimens were autumn spawners; no spring‐spawning herring were identified in the archaeological sites (Figure 2). Archaeological remains from Basel, Switzerland, (Schnabelgasse 6 and Museum der Kulturen, Figure 2) (Häberle and Hüster Plogmann 2019), showed mixed geographical origins, with samples originating from the Baltic and the Atlantic. All other sites exclusively contained samples that were assigned to the modern‐day BINSA metapopulation. Eight archaeological specimens had sufficiently high coverage to conduct DAPC analysis and assess the ability of MSA SNP panels to be used over long timescales. These samples are from the sites of Lyminge in southeastern England (8th–9th century CE) (Reynolds 2013), Coppergate (930–1000 CE) (Bond and O'Connor 1999) and Blue Bridge Lane (13th century) (Keaveney 2005) in York, and Huis in den Struys (15th century) (Laarman and Lauwerier 1996) in the Netherlands. All high‐coverage samples except HuisDenStruys2 were assigned to the winter‐spawning population from the southern and western sides of the UK (Figure 3a). Herring from different spawning populations are observed in the Lyminge and Huis in den Struys assemblages. A higher degree of similarity between Isle of Man and the Celtic Sea/Downs population is exhibited in the ancient samples than the modern samples (Figure 4). The classification based on diagnostic SNPs is consistent across the entire time period studied, with the largest genetic divergence consistently identified between the North Sea population and the other three subpopulations (Jost's *D*, Figure 3b). These results agree with DAPC analysis on the contemporary samples showing two main subpopulations within BINSA: NSAS and southern and western winter spawners with some degree of substructure between Celtic/Downs and Isle of Man. Genetic divergence measured in modern populations alone suggests additional structure between Celtic/Downs and Isle of Man than observed in the past (Figure S13).

### 3.3 Changes in Genetic Diversity Over Time

Ancient samples exhibited larger variation in heterozygosity estimates than modern samples and a slightly higher median heterozygosity estimate per individual when compared to the contemporary samples. HuisDenStruys2 (North Sea) exhibited a significantly higher heterozygosity estimate than the North Sea samples collected in 1979, which in turn exhibited higher heterozygosity than the samples collected in the 2010s (Figure 4c). To account for the potential impact of post‐mortem damage, heterozygosity estimates were calculated using the individual SFS and transitions were removed from analysis. Differences in heterozygosity levels were evaluated using ANOVA in R. Samples were grouped by time period (Ancient, historical, contemporary, where “historical” refers to the samples collected in 1979). Differences between time periods were significant (*p* = 0.033, df = 2) (see Figure S14 for residual plots). Tukey's post hoc analysis suggests there are significant differences between the contemporary samples and ancient samples (*p* = 0.002; see Figure S15), with ancient samples being significantly higher in heterozygosity. The difference between the historical samples and the contemporary samples and between the historical and ancient samples is not significant (*p* = 0.55 and *p* = 0.51, respectively).

ROH were also assessed per population. The contemporary populations exhibited a pattern of ROH that suggests increased inbreeding throughout history, with the majority of individuals exhibiting the highest number of ROH with estimated coalescent ages between 600 and 850 YBP (Figure 4b; Figure S5). The North Sea population diverged from this pattern by exhibiting the largest number of ROH with coalescent ages estimated to 850–900 YBP, a time bin for which the Celtic/Downs and Isle of Man populations exhibit the smallest number of ROH, suggesting a significantly higher level of inbreeding for the NSAS at this time. The only ancient samples with high enough quality for ROH analysis were from Lyminge and had been assigned to separate populations with MSA (Lyminge2, Lyminge3, see Figure 3a) and they could not be used for population‐wide ROH analysis. The fraction of the genome in ROH (F_ROH_) between ancient and contemporary samples was thus compared on an individual basis. Lyminge2 (Isle of Man) and Lyminge3 (Celtic/Downs) were compared with the randomly‐downsampled modern individuals used for the population comparison. Both ancient samples had smaller total lengths of ROH across their entire genomes and a lower fraction of the genome in ROH than the contemporary samples (Table 1). Notably, Lyminge2 exhibited less than half the number and total length of ROH than Lyminge3. No significant relationship between F_ROH_ and sequence coverage was found using linear regression (*p* = 0.62).

**TABLE 1:** Fraction of the genome in a run of homozygosity (*F*_ROH_) by individual for high‐coverage samples.

Sample	# ROH	Total length ROH (Kb)	*F*_ROH_	Coverage
Lyminge3 (Celtic/Downs)	1631	274,637	0.000376	23.4
Celtic2	1774	320,920	0.000440	12.1
Downs1	1740	320,789	0.000439	11
NorthSea1	1726	310,994	0.000417	16.6
NorthSea2	1701	294,174	0.000394	16.4
Lyminge2 (Isle of Man)	399	62,335	0.0000854	11.4
IsleOfMan1	1685	299,837	0.000411	17.2
IsleOfMan2	1703	303,754	0.000416	12.3

### 3.4 Demographic Modeling

When all herring were grouped as a single population, demographic modeling using GONE exhibits two alternating demographic patterns (one with relatively stable population structure over time and one with a large decline and subsequent recovery in the recent past, both characterized by sharp drops in the 5 most recent generations, see Figure S9) indicative of past admixture (Novo, Santiago, and Caballero 2022). GONE is known to be highly sensitive to population structure (Kersten et al. 2023; Novo et al. 2023; Santiago et al. 2020) and, given the time difference in sampling (50 years) between some of the subgroups and the demonstrated substructure within BINSA from DAPC MSA, all four sampling locations were therefore analyzed separately. When run following original metadata (e.g., North Sea, Celtic Sea, Downs, and Isle Of Man), North Sea, Downs, and Celtic Sea population showed signs of admixture (Figure S10). Re‐segregating samples based on their classification with DAPC removed these admixture artifacts, further supporting population assignment results. Downwardly biased estimates for the most recent 4 generations in the Isle of Man and Celtic/Downs estimates suggest ongoing gene flow between these populations, but grouping all three as one population again showed bias due to population structure (Figure S10; Novo et al. 2023). Following Atmore et al. (2022a), these estimates were removed from analysis due to likely statistical bias. Downs4(NS) was removed from this analysis as it was sampled 50 years prior to the remaining Celtic/Downs samples. All BINSA subpopulations showed a similar demographic trend, with the North Sea population showing a larger degree of variation in *N*_e_ estimates across all iterations compared to the other three subpopulations. All three subpopulations show relatively stable population sizes prior to the 20th century, albeit with a slow decline beginning around 1000 CE (Figure 4a). We observe consistent signatures of decline in all three subpopulations beginning in the mid‐20th century, which matches both known commercial collapse (Dickey‐Collas 2016; Dickey‐Collas et al. 2010) and increasing sea‐surface temperature (SST, Figure 4a). No population shows signatures of recovery in recent decades.

## 4 Discussion

We have here demonstrated that the existing population substructure identified among modern herring populations in the North Sea, Channel, and Celtic and Irish seas persists across centuries. Mixed‐stock analysis (MSA) results showing the Dutch herring industry targeted both NSAS and Downs populations suggest mixed‐stock exploitation was a feature of historical fishing operations and is not exclusive to industrial fishing practices. We further find that herring demographic patterns were relatively stable until the dramatic 20th century disruptions. The long‐term persistence of population structure in the Atlantic herring reveals that MSA based on SNP panels is a cost‐effective technique for sustainable management, as it can be applied over long timescales. Recent perturbations in herring demography further indicate that anthropogenic impacts on the species are greater than previously understood. In the following paragraphs, we discuss the novel historical and biological insights, and management implications of our results.

### 4.1 Historical & Biological Implications of Archaeological Population Assignments

Nowadays, the vast majority of modern herring landings in the North Atlantic and the Baltic come from spring‐spawning herring stocks (ICES 2023; MacKenzie and Ojaveer 2018). We discover that all 32 archaeological herring specimens from eight sites across Europe (Norway, Switzerland, the Netherlands, England), spanning a period of 900 years (700–1600 CE) instead consist of autumn spawning herring only. This exclusive dependence on autumn‐spawning herring has also been observed for historic herring exploitation in the Baltic (Atmore et al. 2022a). It is unclear what drives this historic European‐wide pattern of herring exploitation. While it may simply be a cultural preference for the larger, fattier autumn spawners, there is evidence that Atlantic Ocean basins are dominated by one spawning phenotype at a time (Berg et al. 2021); for instance, population declines in autumn spawners resulted in numerical dominance of spring‐spawners in the Baltic (Atmore et al. 2022a; MacKenzie and Ojaveer 2018). It is therefore possible that autumn spawning herring may have been numerically more dominant in the past, and therefore more available to past fishing operations, resulting in their exclusive appearance in the archaeological record. Future studies investigating more archaeological sites, including a higher number of specimens should address this question. Further discussion interpreting results for each specific archaeological site is included in the S1

### 4.2 MSA Across a Millennium

We were able to assign 8 high‐quality (2–23X coverage) ancient genome sequences covering the 8th–15th centuries to local BINSA subpopulations using MSA and found that diagnostic SNPs are robust to long‐term change. Atlantic herring exhibit some migration between stocks, although the level of gene flow and interbreeding co‐occurring with trans‐stock migration is unclear (Berg et al. 2021). We here demonstrate that loci associated with contemporary population structure have changed little over the past millennium, suggesting strong drivers maintaining this structure throughout time, either at these loci or in regions associated with these loci (as constrained by linkage disequilibrium) Combined with the result that only autumn spawners were commercially traded historically, this suggests long‐term population structure and stability in the region. These results also show that the creation of diagnostic SNP panels for MSA is a highly cost‐effective mode of genetic monitoring, as they can likely be used for decades, if not millennia. The loci used for the MSA were composed of SNPs associated with local adaptation, e.g., spawning phenotype and local salinity levels (Farrell et al. 2022a; Han et al. 2020a). Given the shared spawning phenotype and salinity levels experienced by the populations in the Downs and North, Celtic, and Irish Seas, these populations represent an opportunity for future studies to assess fine scale local adaptation beyond the currently recognized metapopulations. We observe limited temporal change in genetic structure over time. Contemporary analyses have shown population structure between Isle of Man and Celtic/Downs populations (Farrell et al. 2022a). In contrast, MSA of archaeological remains and analysis of absolute divergence using Jost's *D* suggests these populations were less differentiated in the past. It is unlikely these results are driven by low sequence quality, as the ancient specimens were filtered for signatures of contamination, low base quality scores, and strict missingness, and transitions were removed to minimize the impact of post‐mortem damage (see Section 2).

Given the known migration patterns of these three stocks (Volkenandt et al. 2015), these results suggest higher levels of gene flow between the Celtic and Isle of Man populations in the past, with Celtic Sea herring as a mediating population for genetic connectivity between Isle of Man and Downs; previous research has demonstrated connectivity between Celtic and Downs populations in the 21st century (Limborg et al. 2012). Such historical connectivity fits our temporal analyses that imply that the contemporary divergence between Isle of Man and Celtic/Downs populations is fairly recent. Atlantic herring are known to exhibit a phenomenon known as the “basin effect,” in which a reduction in population size is associated with retreat to core habitats, and an increase is associated with expanding to new habitats and spawning grounds (Dickey‐Collas et al. 2010; MacCall 1990; Schmidt et al. 2009). Thus, the recent divergence of Isle of Man and Celtic/Downs may be indicative of population decline sometime after the 15th century (the latest date for which we observe admixture between these populations). Indeed, recent evaluation of herring migration patterns in the Celtic Sea concluded that larger population sizes are associated with increased mixing with other stocks and larger migration patterns (Volkenandt et al. 2015). The populations south and west of England should therefore be monitored for overall population health and possible overexploitation.

### 4.3 Reductions in Genetic Diversity and Effective Population Size

The ancient samples largely exhibited higher individual heterozygosity levels and lower F_ROH_ than contemporary samples, suggesting greater genetic diversity and less inbreeding in the past. These patterns are not associated with lower sequencing coverage and are thus not likely driven by technical bias (Kardos and Waples 2024) (see Figure 4b,c; Table 1; Data S1). We further removed transitions from heterozygosity estimation to minimize the potential for post‐mortem damage (PMD) to artificially inflate genetic diversity. Given their lower coverage, heterozygosity estimates for ancient genomes are typically underestimated, suggesting that the true difference in heterozygosity in our samples is likely even larger than reported here. The ancient samples further exhibited a wider variation in heterozygosity and in ROH. Interestingly, the samples collected in 1979 (NS1, NS2, Downs4(NS)) exhibited levels of heterozygosity comparable to those of the ancient samples.

The lack of significant difference in heterozygosity levels between the historical samples and both the contemporary and ancient samples (which were significantly different from each other) is intriguing. The difference between average heterozygosity in the 1979 samples was lower than the ancient samples and higher than the contemporary samples (see Figure S9), and the ancient samples had significantly higher heterozygosity than the contemporary samples, indicating there was a reduction in genetic diversity prior to the 1970s but that reduction was not significant until at least the late 20th century. This result suggests the dramatic stock collapses of the 1960s and 1970s had an enduring evolutionary impact on the Atlantic herring. Given the known impact of low sample sizes and variance in coverage on these analyses (Kardos and Waples 2024), these results must be interpreted with caution; larger studies should re‐evaluate ROH and heterozygosity in the future and further interrogate the timing of increased admixture/heterozygosity. Given the lack of significant difference between the ancient samples and those from 1979, future studies should evaluate the potential of mid‐century samples for providing genetic diversity baseline or threshold values for sustainable management policies.

Modeling of effective population size over time with GONE revealed differentiation between the subpopulations within BINSA, a group that clusters together when conducting structure analysis on the whole genome. GONE is highly sensitive to gene flow and population structure (Novo et al. 2023), and initial results with incorrect grouping of samples revealed signals of admixture and/or population structure (Figures S9 and S10). We detect low levels of gene flow between populations but find that they should not be grouped as metapopulations (Figure S10). This result further supports the biological relevance of the observed population structure between these populations going back through time, particularly between the NSAS and all other BINSA subpopulations. Given the small sample size and possible impact of bias due to gene flow (Novo et al. 2023; Kardos and Waples 2024), we recommend further studies with larger samples to confirm these results. GONE results support the DAPC conclusion that there is a low level of gene flow between Isle of Man and Celtic/Downs populations, but that they should not be considered a metapopulation (Figure S10). Thus, Atlantic herring subpopulations exhibit demographic independence despite known migration between spawning populations (Berg et al. 2021), highlighting the importance of monitoring these populations as separate management units (Bekkevold et al. 2023).

A recent reevaluation of herring landings across all North Atlantic fisheries since 1500 suggests that catches across all populations hovered around 100,000 t per annum before dramatically growing in the 18th–19th centuries (Holm et al. 2022). While fishing pressure across the board was thus relatively constant, pressure on specific stocks/populations shifted dramatically over time. Previous studies on the impact of historical fisheries in the North Sea have suggested several periods of low herring recruitment between 1500 and 2015 were associated with declines in primary productivity (Estrella‐Martínez et al. 2019; Scherer et al. 2024). These fluctuations are not directly reflected in our models of effective population size over time; *N*_e_ estimates often smooth these relatively short‐term census fluctuations (Hare et al. 2011). We do see, however, a slow decline in *N*_e_ starting around 1000 CE for the NSAS, which could be due to the combined impact of warmer temperatures (Estrella‐Martínez et al. 2019; Mann et al. 2009; Ojaveer et al. 2015; Polte et al. 2014) (but see Ojaveer et al. (2024)) and increasing exploitation (Kowaleski 2016; Poulsen 2008). Intriguingly, this decline slows to seeming stability for NSAS and Isle of Man during the Dutch Herring industry and is not mirrored in the Celtic/Downs admixed population, perhaps due to a larger population size and increased gene flow between the two spawning aggregations.

We observe declines in *N*_e_ from five to four million, with one period of slow decline occurring around 1000 CE and a recent period of rapid decline in the last 50–70 years. While this decline appears to be of limited impact—an *N*_e_ of 4 million and a likely low *N*_e_/*N* ratio suggests herring populations are still enormous—we consider this decline in historical context. First, the most significant portion of this reduction occurred in the past 70 years, representing a near‐20% reduction in effective population size. This non‐trivial change in herring effective population size may have lasting impacts on its resilience and capacity for recovery. Indeed, herring sampled in 1979—which may have lived through the commercial collapse—had levels of genetic diversity comparable to the archaeological samples and higher than contemporary samples (Figure 4c). While the metapopulation recovered in terms of SSB in the early 2000s, spatially disparate patterns between the North Sea spawning populations meant the stock did not exhibit full recovery at any point after the 1970s collapse (Schmidt et al. 2009). For those populations in our study with samples postdating the recovery period (Celtic/Downs, Isle of Man), we see no recovery in effective population size. Indeed, our discovery of recent divergence between Celtic/Downs population and the Isle of Man population suggests there has been a strong evolutionary impact from the decline through reduced gene flow between these populations, a result supported by our GONE analysis of population groups (Figure S10). The lack of admixture discovered in these populations in contemporary samples indicates ongoing evolutionary consequences for the Atlantic herring from the overfishing in the 20th century.

Prior studies on the impacts of fish stock declines, such as the notorious collapse of Grand Banks cod industry in Newfoundland, have found little‐to‐no effect on genetic diversity (Pinsky et al. 2021), although other studies imply that genetic diversity in marine fish stocks can be lost without parallel census population decline (Hauser et al. 2002; Hutchinson et al. 2003). Small pelagic fish, while seemingly inexhaustible, are known to be vulnerable to overfishing (Durant, Holt, and Langangen 2024; Langangen and Durant 2024; Ottersen et al. 2013). Further, modeling has shown that small pelagic fish are more vulnerable to environmental changes after exploitation‐driven stock collapses (Durant, Holt, and Langangen 2024). The observed reduction in *N*_e_ in the past 50–70 years is thus likely due to the combined and ongoing stressors of warming temperatures, shifting ocean currents, and overexploitation. The dramatic evolutionary consequences highlight the importance of incorporating long‐term fishing records and modeling into management advice; allowing a stock to recover to an arbitrary, post‐exploitation biomass threshold over the course of a few strong year‐classes may not be sufficient to ensure long‐term sustainability of these stocks.

### 4.4 Addressing Limitations

This study relies on a relatively small dataset. This is partially due to the limitations of working with ancient specimens, and the poor availability of modern sequencing data (genome sequences and/or appropriate metadata) of herring from prior studies. Nonetheless, despite the small sample size, we genetically identify known population declines (e.g., 20th century commercial collapses), are able to correctly assign contemporary samples to their known population of origins and recover fine scale population structure using the same loci used for contemporary fisheries management (Bekkevold et al. 2023; Farrell et al. 2022a). Although, it should be noted that Celtic Sea and Isle of Man populations were the least‐represented samples in the creation of the SNP panels for MSA. Studies incorporating more contemporary and ancient samples from these populations would greatly strengthen the result by increasing power to differentiate these populations and confirm our reported results.

One final concern when dealing with such a study is the question of independent sampling when comparing archaeological specimens to their modern conspecifics. For population genomics studies, non‐independent observations may be obtained by sampling closely related individuals or due to closely related segments of the genome (Waples, Waples, and Ward 2021). We tested genetic relatedness of all individuals using KING (Manichaikul et al. 2010), determining that none of them were significantly closely related and are thus independent observations. Historical fishers were fishing from the same biological populations that are under exploitation today, and it is known that they targeted spawning aggregations of mature adults in such quantities that they are comparable to industrial‐scale harvesting (Holm et al. 2022; Poulsen 2008). As a valued commercial product, herring were traded between the archaeological sites investigated here, thus our samples do not merely reflect small local populations and are comparable to contemporary samples. Further, studies modeling the impact of non‐independent observations when conducting whole‐genome sequencing have suggested that larger numbers of loci and access to a high‐quality reference genome greatly reduces the likelihood of bias from non‐independent observations, even with extremely small sample sizes (Waples, Waples, and Ward 2021). This study is therefore unlikely to reflect such bias due to the large number of loci under consideration (~10 million) and the use of a high‐quality reference genome.

### 4.5 Long‐Term Viability of Genetic Tools for Management

We here demonstrate both the long‐term potential of panels developed for MSA of the Atlantic herring and significant changes in herring demography within the last century. Future research efforts with larger, more comprehensive datasets will be able to confirm this pattern, provide more power for discriminating between closely related subpopulations (e.g., Downs and Celtic Sea), and further explore issues such as exact time of divergence and causal ecosystem dynamics. For example, it has been suggested that Atlantic cod and Atlantic herring do not exist at high biomass at the same time (Dickey‐Collas et al. 2010)—ancient DNA and genomic modeling on cod and herring simultaneously could reveal shifting stable states in the North Sea between these two economically important species. Better understanding the dynamics at play in the North Sea ecosystem will provide a more solid foundation for developing sustainable management regimes and conservation strategies, such as ensuring ecosystem resilience and the continued survival of fisheries and other species that rely on the Atlantic herring (Fayet et al. 2021).

Our results suggest strong perturbations in herring population genetics in the 20th century, including likely impacts of climate change and overfishing. Increased admixture between populations in the past is likely due to larger population sizes throughout history compared to modern‐day herring stocks, a hypothesis that is supported by the recent population declines recovered through demographic modeling in this study. We find sharp decreases in effective population size in herring populations in the 20th century at a time when overfishing is known to have caused commercial stock collapse in the region. Despite reported increases in census stock size after a brief moratorium on these stocks, effective population size has not recovered for the Atlantic herring stocks here assessed and, indeed, continues to decline. These observations suggest these populations are not recovering from the 20th‐century stock collapses, which could be due to continued overfishing and the effects of warming sea‐surface temperatures. The genetic diversity results further imply there is a practical 20th genetic population “baseline” for herring in the North, Celtic, Irish, and Downs Seas (although see Atmore, Aiken, and Furni (2021) for a critique of the baseline concept). Future studies querying the existence of admixture between herring stocks in more recent time periods are warranted to support this conclusion. We further demonstrate the Atlantic herring exhibit strong population structure that dates back at least 1300 years and can be identified from ancient specimens using the same diagnostic SNP panels that have been developed for contemporary populations. Divergence analysis indicates loci identified for contemporary MSA have changed remarkably little over the past millennium, suggesting strong pressure on these sites to maintain local adaptations and population structure. These results show that the development of diagnostic SNP panels is a highly cost‐effective measure for genetic monitoring of herring stocks.

## Author Contributions

**Lane M. Atmore:** conceptualization, data curation, formal analysis, investigation, methodology, software, visualization, writing – original draft, writing – review and editing. **Inge van der Jagt:** investigation. **Aurélie Boilard:** investigation. **Simone Häberle:** investigation. **Rachel Blevis:** investigation. **Katrien Dierickx:** investigation. **Liz M. Quinlan:** investigation. **David C. Orton:** investigation. **Anne Karin Hufthammer:** investigation. **James H. Barrett:** funding acquisition, project administration, supervision, writing – review and editing. **Bastiaan Star:** conceptualization, funding acquisition, project administration, resources, supervision, validation, writing – review and editing.

## Conflicts of Interest

The authors declare no conflicts of interest.

## Supporting information


Appendix S1.

Data S1.

Data S2.

## Data Availability

The data that support the findings of this study are openly available in Zenodo at https://zenodo.org/10.5281/zenodo.14366787 and the European Nucleotide Archive under project PRJEB77597. The code that support the findings of this study are openly available in Zenodo at https://doi.org/10.5281/zenodo.14427350 and GitHub at https://github.com/laneatmore/OnceandFutureFish/tree/main. Additional genomic sequence data were obtained from the European Nucleotide Archive under projects PRJEB52723 and PRJNA642736.
