# Supplementary material for: The Once and Future Fish: Assessing a Millennium of Atlantic Herring Exploitation Through Mixed‐Stock Analysis and Ancient DNA
Source: Glob Chang Biol. 2024 Dec 26;30(12):e70010. doi: 10.1111/gcb.70010 (PMC11670043; doi:10.1111/gcb.70010)
Supplement: Supplementary file 1 — Appendix S1. [file GCB-30-e70010-s002.docx]

**Supplementary Material**

**The Once and Future Fish: assessing a millennium Atlantic herring exploitation through mixed-stock analysis and ancient DNA**

Lane M. Atmore^1,2*^, Inge van der Jagt^3^, Aurélie Boilard^1^, Simone Häberle^4^, Rachel Blevis^5^, Katrien Dierickx^6,7^, Liz Quinlan^7^, David C. Orton^7^, Anne Karin Hufthammer^8^, James H. Barrett^6^, Bastiaan Star^1*^

1. Centre for Ecological and Evolutionary Synthesis, Institute of Biosciences, University of Oslo
2. Lab of Archaeology, Department of Anthropology, University of British Columbia, Vancouver, Canada
3. Cultural Heritage Agency of the Netherlands, Amersfoort, Netherlands
4. Integrative Prehistory and Archaeological Science, Department of Environmental Sciences, Basel University, Basel, Switzerland
5. Department of Archaeology, University of Cambridge, Downing Street, Cambridge, CB2 3DZ, UK
6. Department of Archaeology and Cultural History, NTNU University Museum, Norwegian University of Science and Technology (NTNU), 7012 Trondheim, Norway
7. Department of Archaeology, University of York, Heslington YO10 5DD, York, UK
8. Department of Archaeology, University of Bergen

**This Supplement Includes:**

Detailed assignment results from archaeological sites (this document)

Supplementary figures S1-S15 (this document)

[Table S1](https://docs.google.com/spreadsheets/u/1/d/10VW0fxdSHMATiz9ewU5aDoQHFsZySjzC_4JB-Oj25jU/edit) – Archaeological metadata

[Table S2](https://docs.google.com/spreadsheets/u/1/d/1CQxL18DziG_GG0THVacQYl24QHMW4Jfd1HWDrGKo3_g/edit) – Contemporary metadata

Diagnostic_sites.list – SNPs used for mixed-stock analysis

NorthSea_reconstructions.txt – SST reconstructions

Works Cited (this document)

**Detailed Assignment Results for Archaeological Sites**

Switzerland

Basel comprises two archaeological urban sites: Schnabelgasse (12th century), and Museum der Kulturen (15th-16th centuries). Basel was at the time, a distribution center for commodity goods such as herring (Jahnke, 2000). The majority of these specimens stem from the Baltic, likely traded from the Øresund industry and its successors (Atmore et al., 2022). There are various trade routes these specimens could have taken, including being imported to England and then re-exported to the continent, or through trade routes along the Rhine (Jahnke, 2000). Genome sequence quality of samples assigned to BINSA was not high enough to identify their subpopulation. Given the known long-distance trade from southern England to France and Germany (Kowaleski, 2016), it’s possible the BINSA remains stem from fisheries along the south or east coast of England. This origin is particularly likely for the remains stemming from the 12th century, at which time herring was traded from southern England to the continent for wine from France (Barrett, 2016), and as the Dutch herring industry was not yet dominant (Poulsen, 2008). The samples recovered in Basel from the 15th-16th century, however, likely do stem from the North Sea autumn-spawners and the Dutch herring industry. Overall, the herring remains from Basel highlight the overwhelming presence of autumn-spawning herring in the commodity fish trade during the medieval era.

Norway

Kaupang is one of the earliest Viking urban centers in Scandinavia, dating to the 9th century. It is located in southern Norway on the Skagerrak (Skre, 2007). Today, herring in the Skagerrak consist almost entirely of Norwegian spring-spawners and western Baltic spring-spawners; while NSAS are today sometimes found in the Skagerrak, they do not appear in large, coastal aggregations (Poulsen, 2010). Thus, the identification of the Kaupang herring sample as an autumn- rather than a spring-spawner is unexpected. NSAS coastal aggregations in the Skagerrak have appeared periodically throughout history during the so-called “Bohuslän periods,” in which a large, overwintering stock (Corten, 1999) flooded the Skagerrak such that they could be harvested at scale from the beach (Alheit & Hagen, 1997; Höglund, 1972). These periods occurred nearly every century between the 11th and 20th centuries, likely driven by cyclical changes in the North Atlantic Oscillation (NAO) (Corten, 1999). So-called “high” NAO periods cause shifts in current and wind speed resulting in colder temperatures and an influx of North Sea waters (and herring) to the Skagerrak. Outside of Bohuslän periods, which typically lasted 20-50 years, NSAS did not appear in the Skagerrak in large enough numbers to approach the coast, and fisheries that sprang up alongside the Bohuslän phenomenon would collapse (Corten, 1999; Höglund, 1972). Contemporary NSAS overwinter in the northeastern North Sea on the opposite end of the Norwegian Trench from the Skagerrak (Corten, 1999). This region is also where the Dutch herring industry later started their herring fishing, near Shetland where the largest concentration of herring was to be found prior to- and during their spawning season (Poulsen, 2008, 2010). The existence of a NSAS herring in 9th-century Kaupang *prior* to written records thus suggests the Bohuslän herring phenomenon may have occurred at least two centuries earlier than previously known.

England

The site of Lyminge was an Anglo-Saxon monastery in present-day Kent dating to the 8th century and is associated with the early medieval revival of marine resource consumption in England, which had largely vanished during the earlier Anglo-Saxon period (Galloway, 2017). Our detailed assignment results of herring remains at Lyminge resulted in a mixed signal between Celtic, Downs, and Isle of Man (Figure 3a). Historical evidence suggests herring at Lyminge stem from local populations and were used to feed the religious brethren at the monastery and/or to pay tithes and rent (Galloway, 2017). Given the evidence for recent population decline and the known phenomenon of the basin effect in Atlantic herring, the presence of mixed genetic ancestry in Lyminge herring supports our conclusion that herring populations around the UK and Ireland were larger in the past, thus exhibiting a higher degree of genetic connectivity.

Coppergate (10th-11th centuries) is an assemblage from York during the Anglo-Scandinavian Age (Bond & O’Connor, 1999; Hall, 1994). While most of the archaeological specimens could only be assigned to the BINSA metapopulations, several were of high enough quality for detailed assignment, resulting in assignment to mixed origins, including both Celtic/Downs and Isle of Man (Figure 3a). During this period, there is strong evidence of trade between Dublin, the “Southern Isles” (inc. Isle of Man), and York (Jorvik) (Hall, 1994; Horne, 2021). The identification of “Downs” specimens in Coppergate suggest these samples could stem from the East Anglian fishery. East and south coast English herring fisheries are known to have expanded in the 11th century, and historical and archaeological evidence suggests these fish were likely traded across England (Barrett et al., 2004; Serjeantson & Woolgar, 2006). Our genetic results therefore agree with historical sources, but whether the remains from Coppergate were sourced from a single source population, stem from both southern England and Dublin, or changed trade origin over time is a question for future studies with increased power to discriminate between Celtic Sea and Downs populations.

Blue Bridge Lane (BBL) is also an assemblage from York and is dated to the 14th century. These samples come from a single pit full of herring, thus are presumed to represent a single-origin herring shipment (Harland et al., 2016; Keaveney, 2005). Detailed genetic assignment of remains from BBL shows that the sample is most related to contemporary Isle of Man (Figure 3a), yet analysis of divergence between temporally disparate populations suggest it is actually closer to an admixed Celtic/Downs population or the NSAS than contemporary Isle of Man (Figure 3b). Archaeological analysis suggests these remains were not processed like medieval Øresund herring catches; thus they may be from Irish or English fisheries (Harland et al., 2016; Keaveney, 2005). At this time, the southern and eastern English fisheries were dominant players in the regional herring market. Fishing towns in Suffolk — one of the counties in East Anglia — dominated the English herring industry in the 10th-14th centuries; Yarmouth, Ipswich, and Dunwich (a now-submerged town) traded fish across England, France, and Germany (Kowaleski, 2016). These towns – Dunwich in particular – are well within current spawning grounds for the Downs population (Daan et al., 1990; Kowaleski, 2003) and would have targeted coastal spawning aggregations. However, given the high degree of similarity between NSAS and the BBL sample, it is not possible to preclude that this sample stemmed from a local Yorkshire fishery targeting NSAS. Future research on more samples from this site would provide additional clues to specific origin, but our results provide novel genetic evidence (Fig 2) to support the archaeological analysis indicating these samples do not stem from the Øresund herring fishery.

Netherlands

Huis in den Struys is a site associated with a trading center that started operations in the 13th century in what is now known as Veere, a town in the Zeeland province of the Netherlands (Laarman & Lauwerier, 1996). Zeeland was known to be a major region practicing distant-water herring fishing for the Dutch Republic by the 15th century (Sicking & Abreu-Ferreira, 2009). The remains are thought to be dated to the 15th century, given that they have been processed for packaging in barrels, which was not locally adopted earlier (20). The Dutch Republic targeted mainly the spawning aggregation on the Fladen Ground off Scotland but was also known to target the Downs population towards the end of the fishing season from the 14th-17th centuries (Kowaleski, 2003; Poulsen, 2008). Our detailed assignment of one sample from Huis in den Struys to the Celtic/Downs population provides novel genetic evidence for the Dutch herring industry targeting multiple biological populations. These results highlight the importance of population assignment for modeling historical catch records; the Downs population is clearly genetically distinct from NSAS and has been for over 1000 years. Thus, models using catch records to estimate historical landings must take herring population structure into account to accurately determine historical impacts such as overfishing rather than assuming that all North Sea herring fisheries would have targeted a single, panmictic population.

**Supplementary Figures**

**
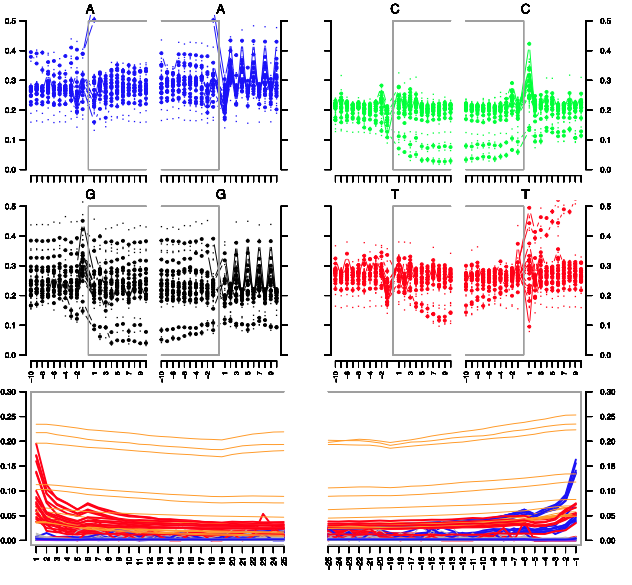
**

**Figure S1 – mapDamage plots for ancient individuals.** Ancient samples exhibited classic signs of postmortem damage, which validates their interpretation as ancient samples. Those samples exhibiting contamination were removed from analysis.


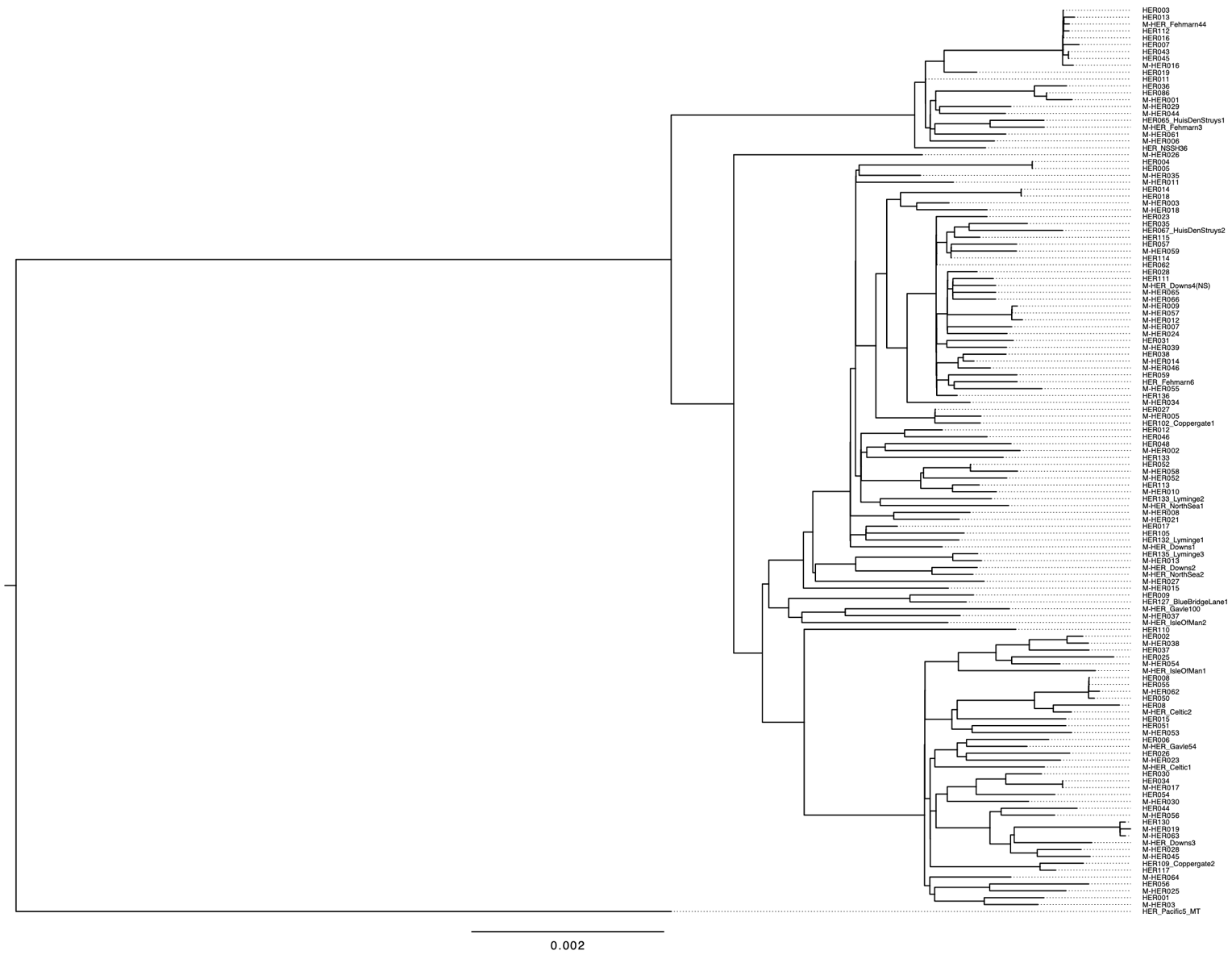


**Figure S2 – Maximum Likelihood Tree for Herring Mitogenome.** Constructed using IQ-Tree with default settings and a Pacific herring (*Clupea pallasii*) as the outgroup, this tree confirms the ancient samples fall within the diversity of Atlantic herring. No mitogenome structure corresponding to geography is exhibited. Contemporary samples are labeled with the prefix “M-HER”; ancient and modern samples interrogated with DAPC are labeled with the same names as are used throughout the paper. This tree was constructed with unique sequences only, thus samples that are directly interrogated in the paper and had identical sequences in the dataset were preferentially included.

**a)**


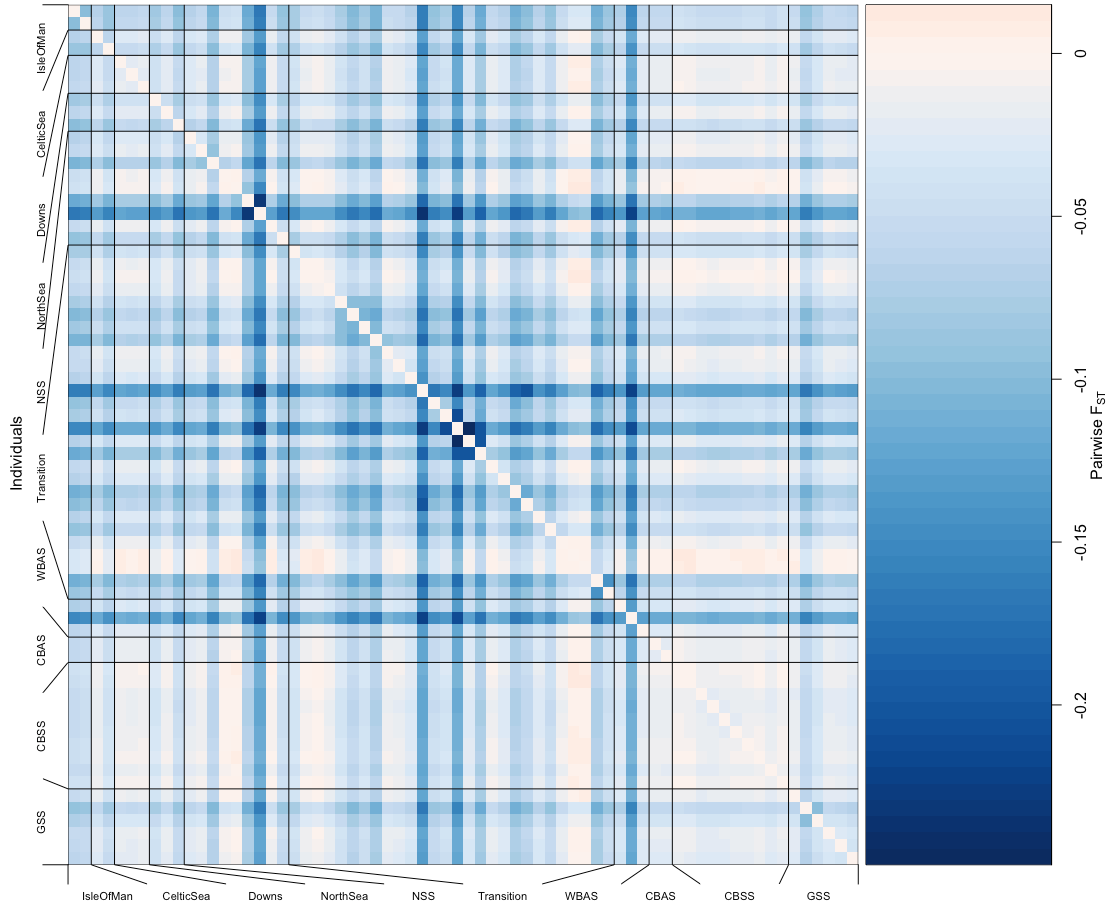


**b)**


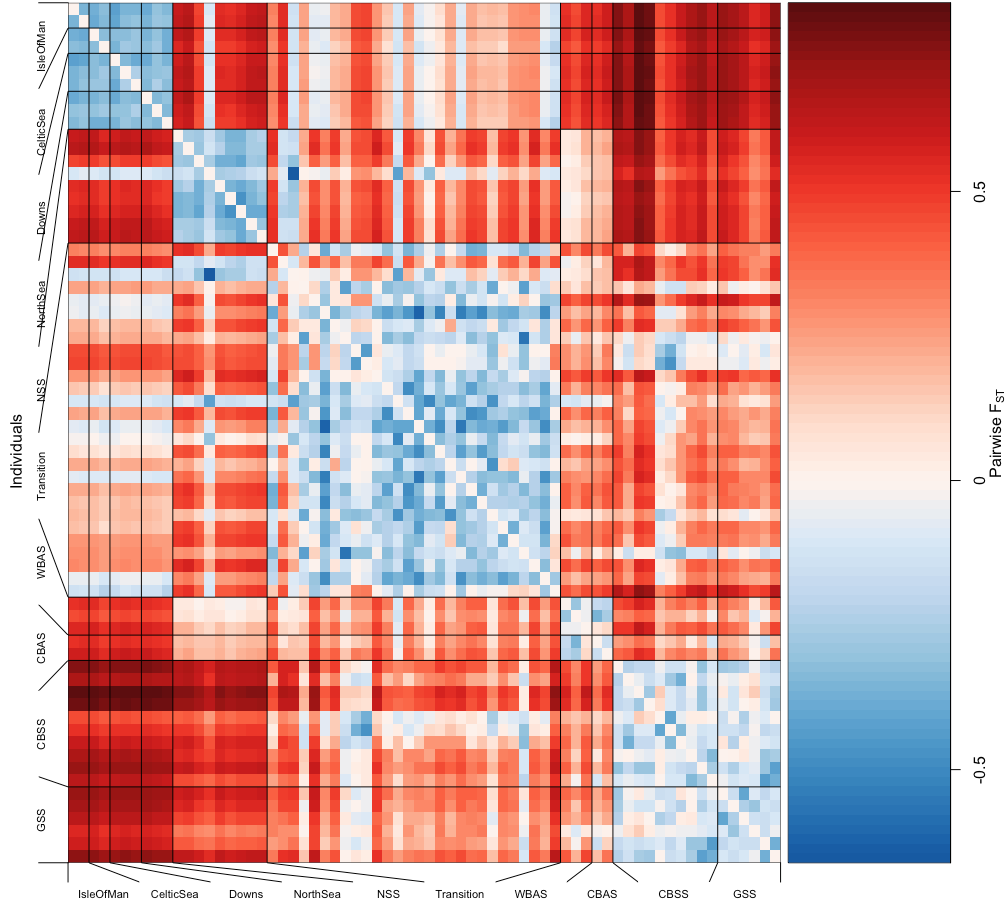


**Figure S3 – Pairwise Fst with Popkin.** a) Hudson’s pairwise F_ST_ calculated with neutral SNPs (~4 million, maf-filtered and pruned for LD) from 68 contemporary herring samples across eastern Atlantic and Baltic. Individual pairwise Fst estimates show zero or negative F_ST_ values, indicating no population structure across all Atlantic herring populations in this study; b) Hudson’s pairwise F_ST_ with SNPs identified as outliers with PCAdapt. Negative or zero F_ST_ values are here restricted to within-group variation, while between group variation is delineated based on spawning season and adaptation to salinity reflecting known population structure.

**a)**
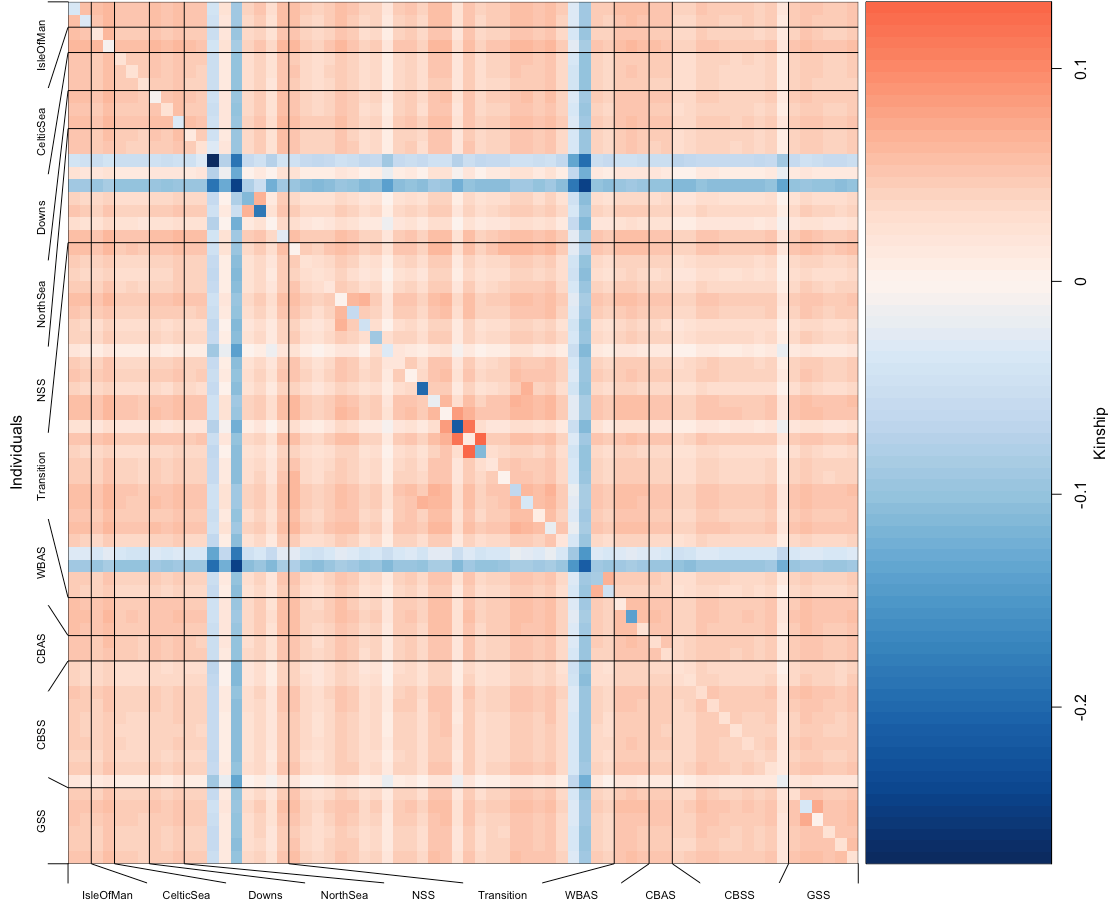


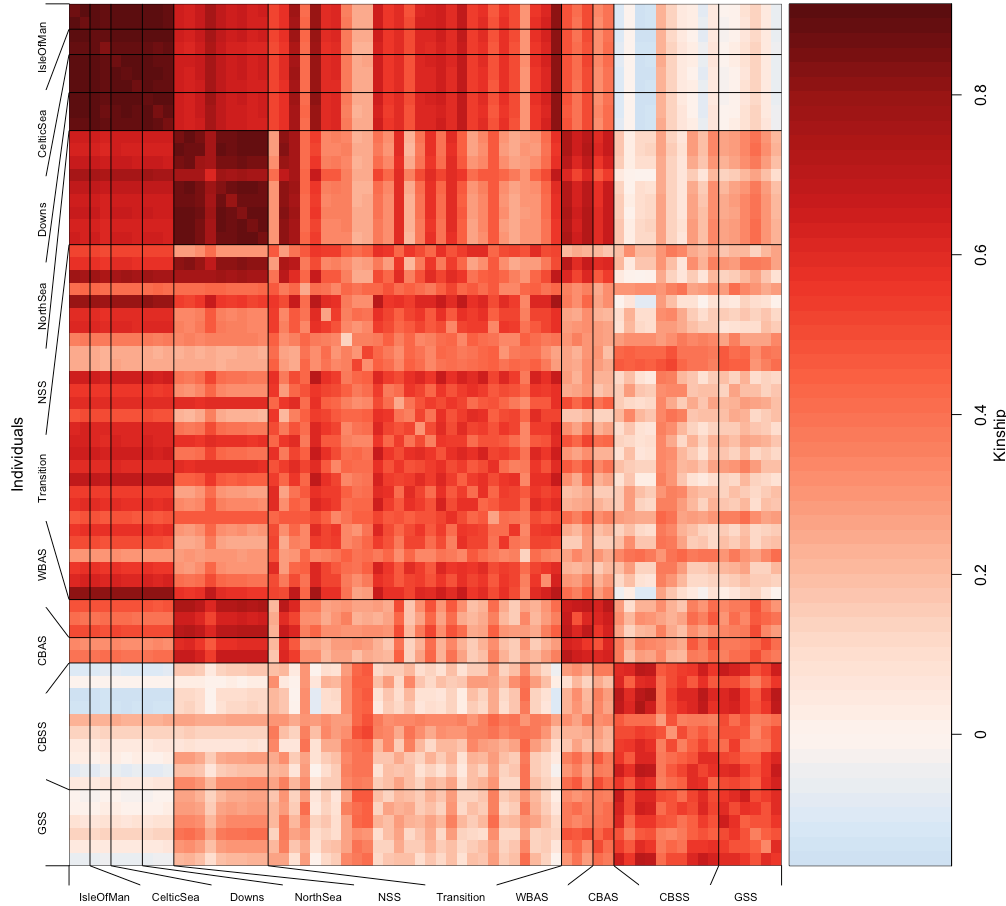


**b)**

**Figure S4 – Pairwise individual kinship matrix.** The kinship matrix was calculated using popkin on 68 contemporary herring samples from across the eastern Atlantic and the Baltic. a) ~4 million neutral SNPs (MAF-filtered, LD-pruned) fail to recover any population structure across these metapopulations; b) SNPs identified as outliers by PCAdapt show increased kinship within known metapopulations delineated by salinity adaptation and spawning season.

**
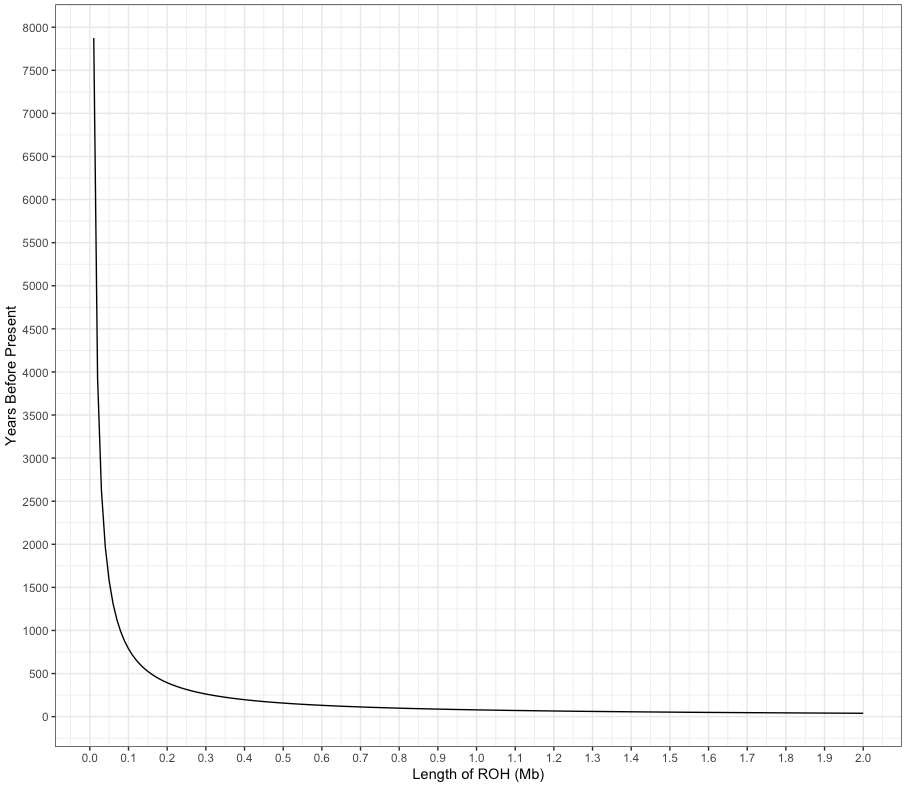
**

**Figure S5 – Time to coalescence for length of Run of Homozygosity.** Estimated using the formula 100/2g cM/Mb=L and herring recombination rate of 2.54 cM/Mb (Pettersson et al., 2019) and generation time of 4 years.

**
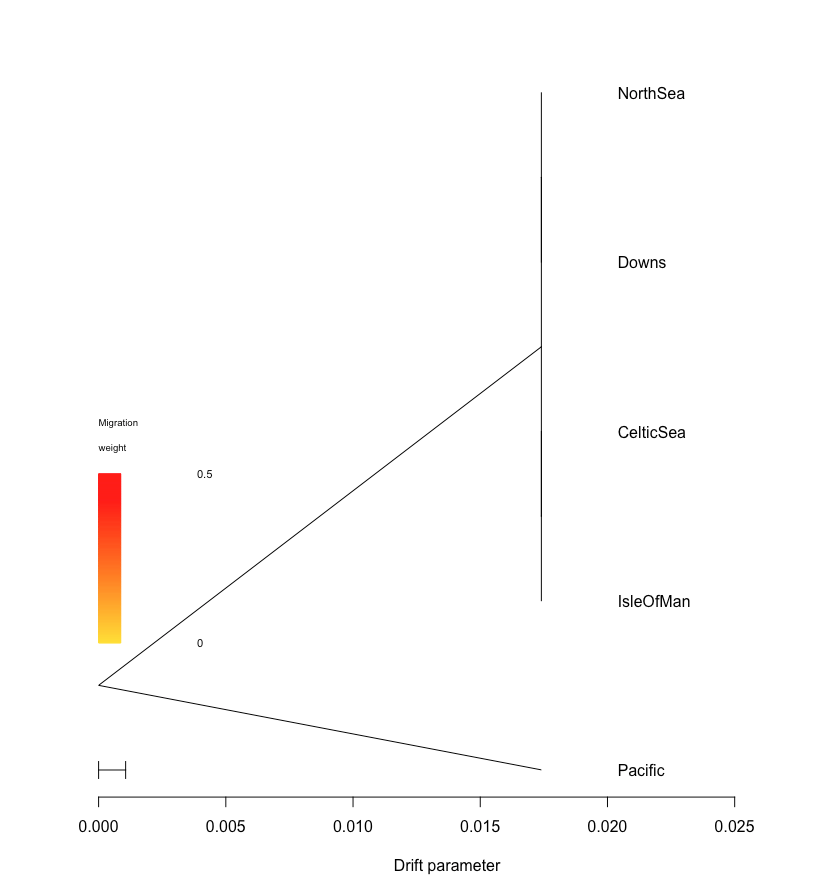
**

**Figure S6 – Treemix results for LD-pruned whole-genome dataset** on contemporary samples reveals no population structure between the BINSA populations.


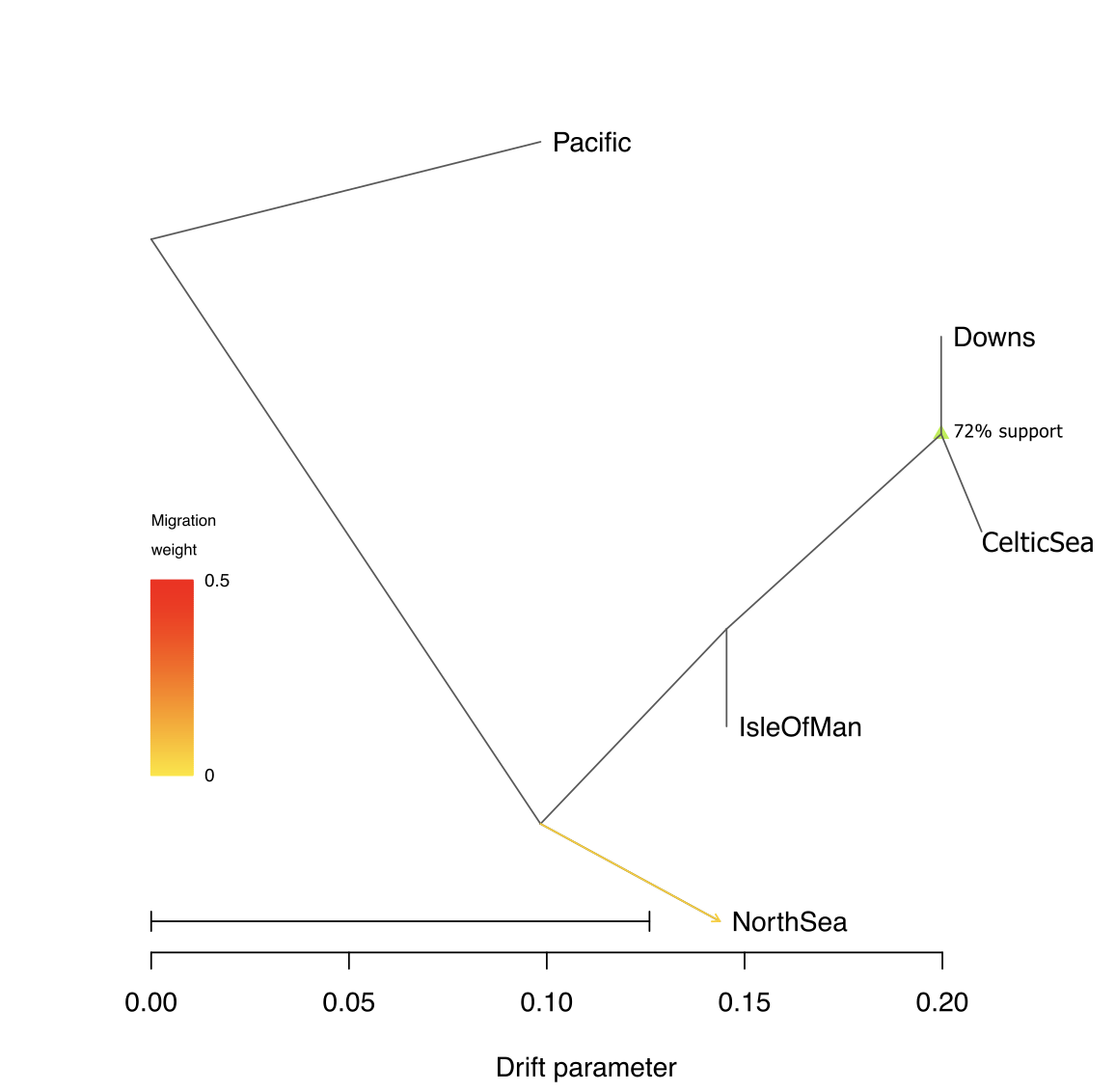


**Figure S7 – Treemix with 1 migration edge for MSA diagnostic SNPs** on contemporary samples suggests separation between North Sea and Downs/Celtic/Irish seas, with Downs and Celtic Sea being most closely related. The single migration edge appears from the common ancestor of the western BINSA populations to the North Sea. All nodes have 100% bootstrap support except the split between Downs and Celtic, which has 72% support.

**
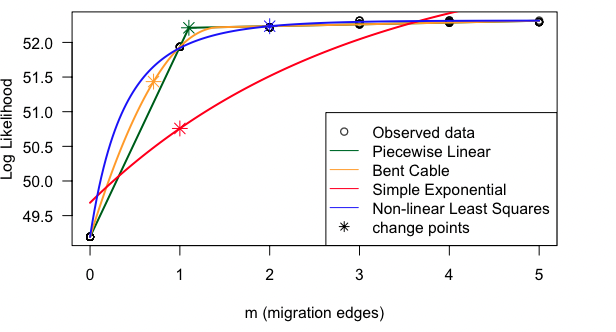
**

**Figure S8 – OptM results** suggesting one migration edge is appropriate for the MSA diagnostic SNP data


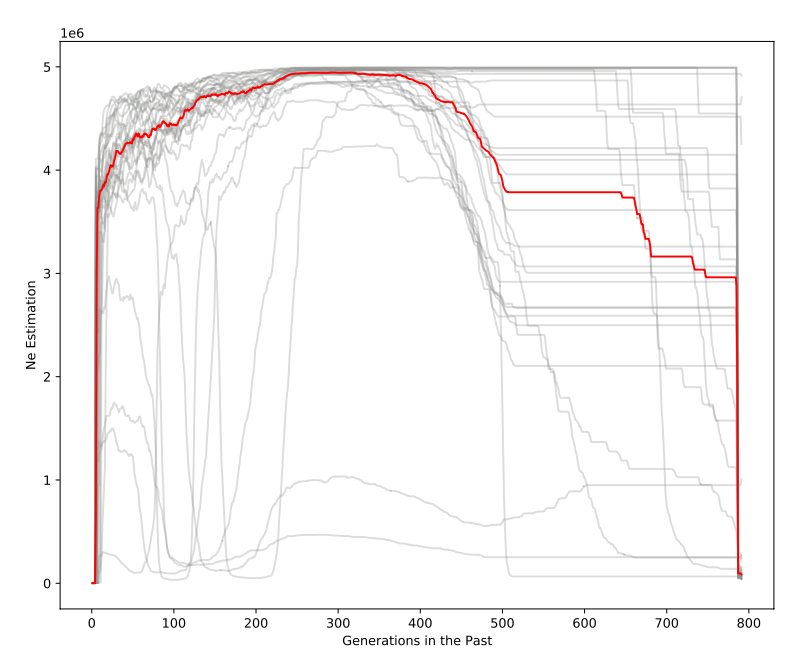


**Figure S9 – GONE on BINSA metapopulation combined** shows many different population trajectories for each iteration, indicating possible population structure confounding the signal.

**Figure S10 – GONE run on BINSA subpopulations to determine appropriate population groupings.** GONE results were interpreted as follows according to results from Novo et al. (2023): The dramatic decline and subsequent recovery trajectories (panels a,b,c,g) are indicative of historical divergence, suggesting there is population structure biasing the results from these groupings. This includes the North Sea population with the individual identified as Downs (a); the Downs population on its own, likely showing the impact of admixture with Celtic Sea (b); and the Downs population with the historical sample from 1979 (c).  Initially distinct yet converging trajectories with downwardly-biased recent generations imply low levels of gene flow detected for Celtic Sea, Celtic/Downs, and Isle of Man (panels d,e,f), reflecting the DAPC results that gene flow did occur between these groups, perhaps as recently as the early 20th century. When grouped together, the trajectory from Isle of Man, Downs, and Celtic Sea shows signs of bias from historical divergence (g) suggesting the gene flow is not considerable enough for them to be grouped as a single metapopulation. This further reflects the DAPC results from contemporary samples that suggests population structure is extant between these groups.


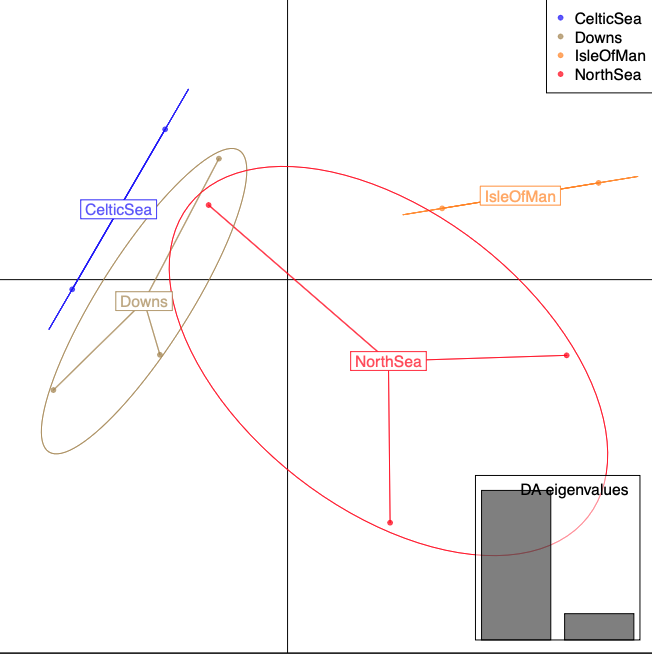


**Figure S11** – **PCA produced with DAPC** suggesting one individual from the North Sea population clusters with the Downs population using SNPs designed to discriminate between Downs and North Sea herring (24).


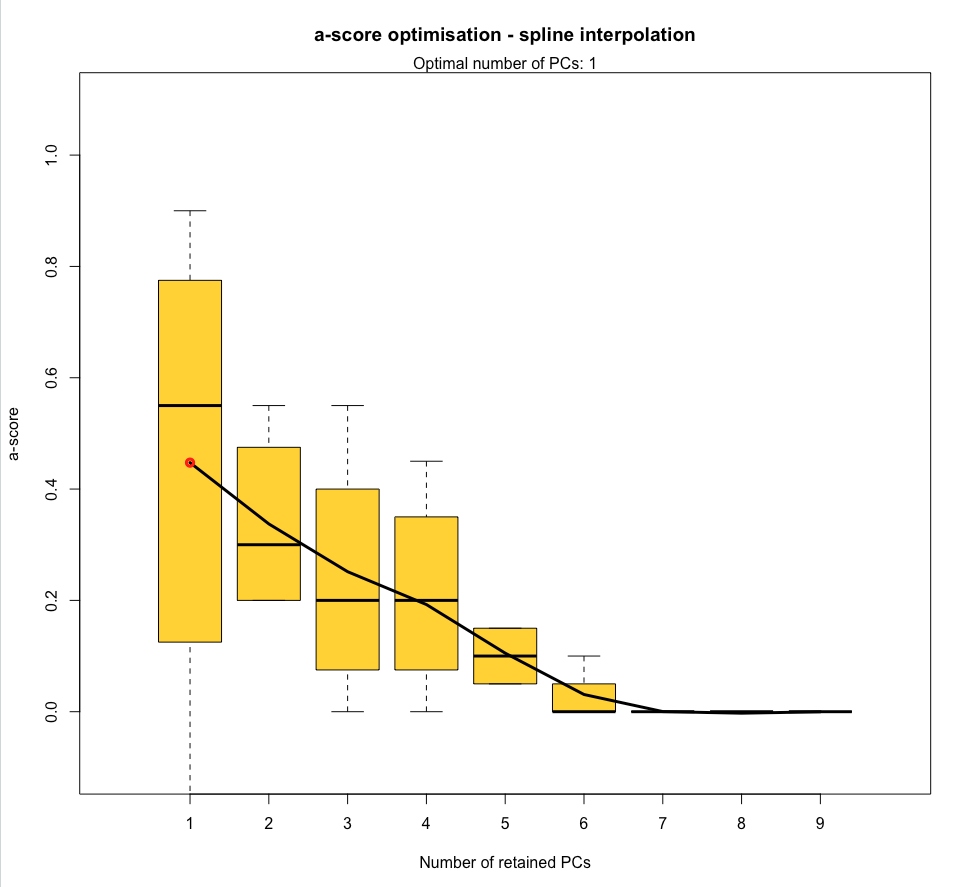


**Figure S12 – Spline analysis for optimal number of PCs to retain for DAPC analysis.** Results suggest using one PC for discriminant analysis.


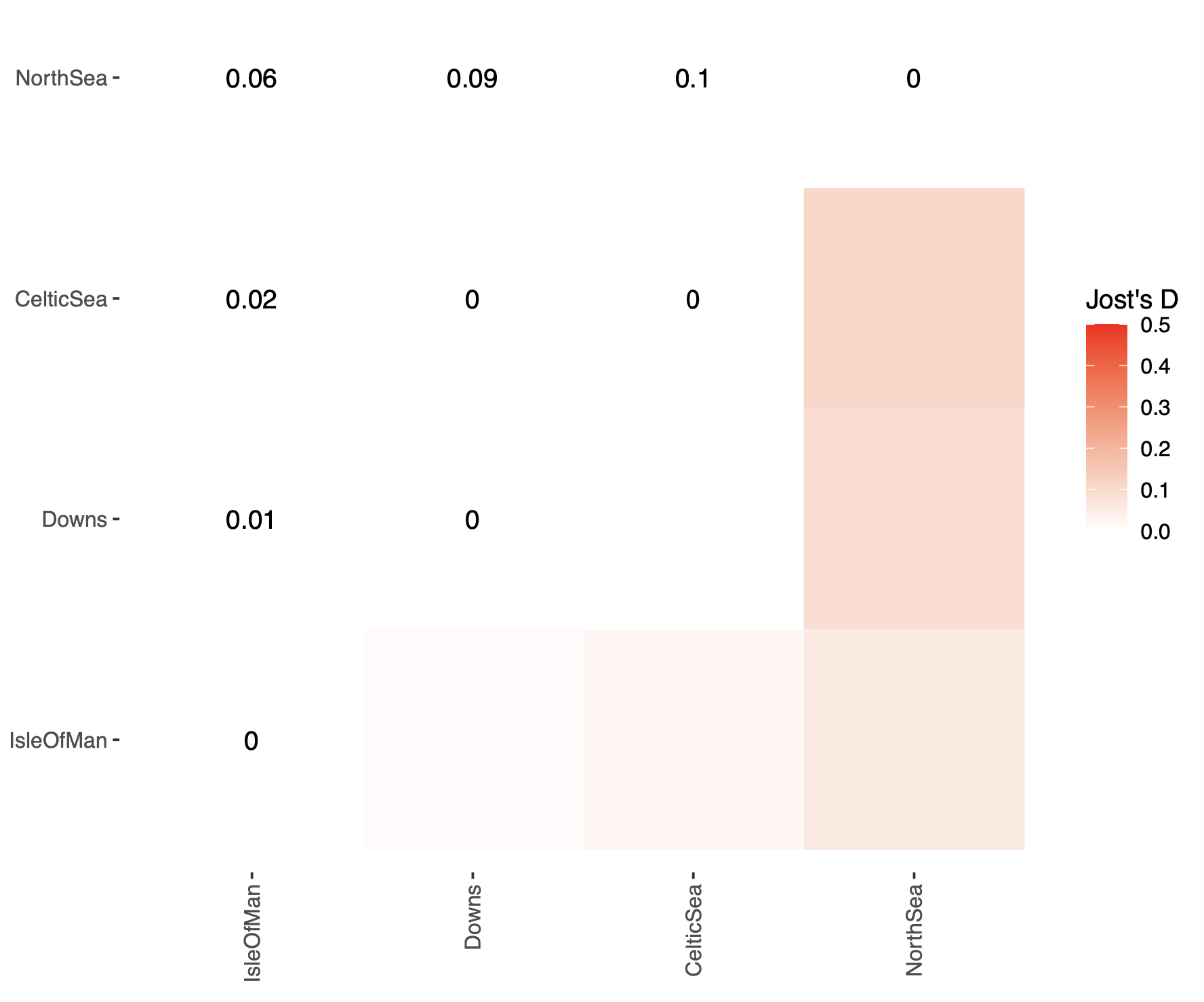
**Figure S13 – Jost’s D analysis of divergence between modern BINSA subpopulations** showing no differentiation between Downs and CelticSea and some variation between this population and Isle of Man. The majority of differentiation occurs between the North Sea and all other populations.


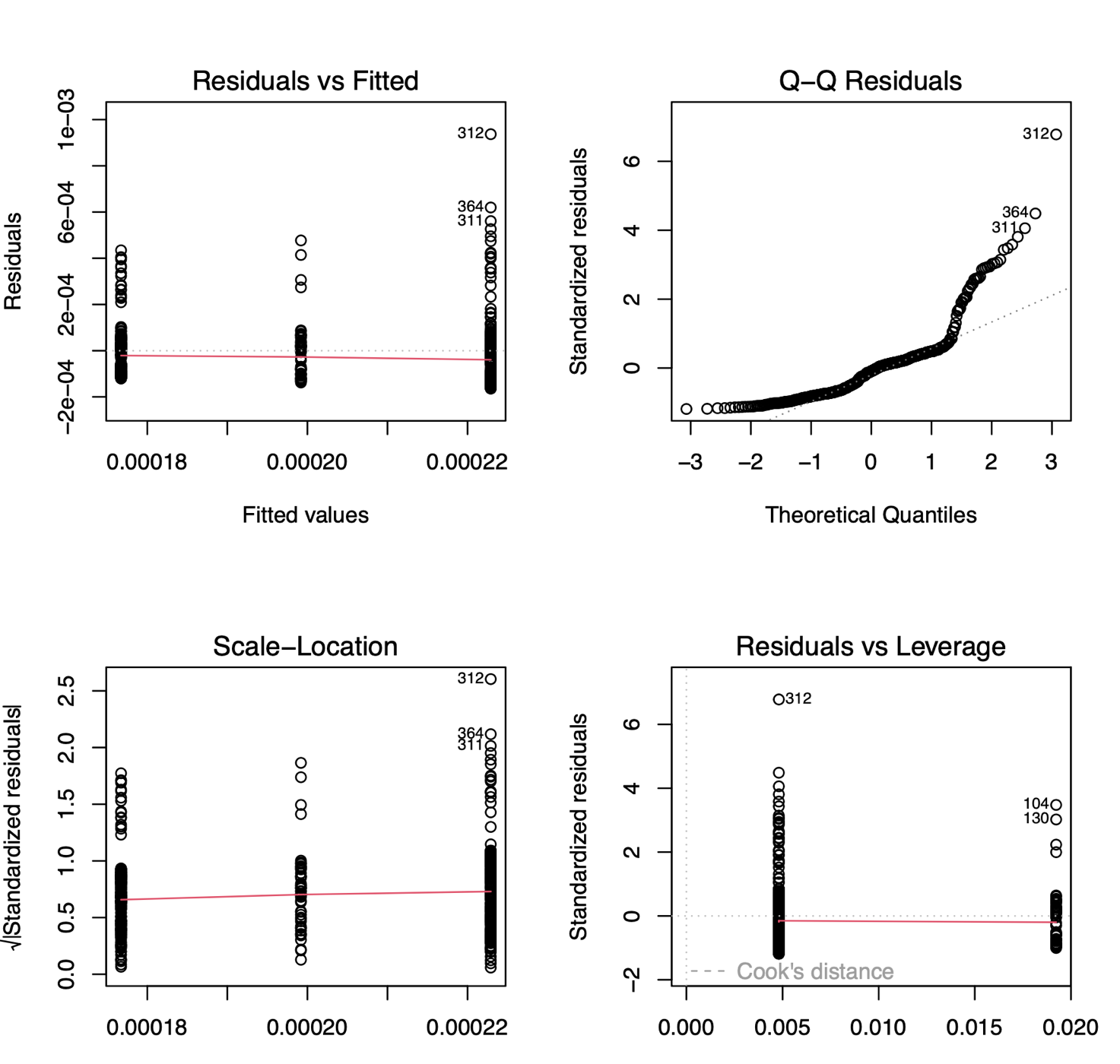


**Figure S14 – Residual values for one-way anova evaluating differences in heterozygosity levels based on time.** Residuals suggest the ANOVA test is appropriate for this dataset, as the data largely conforms to expectations of heteroscedasticity.


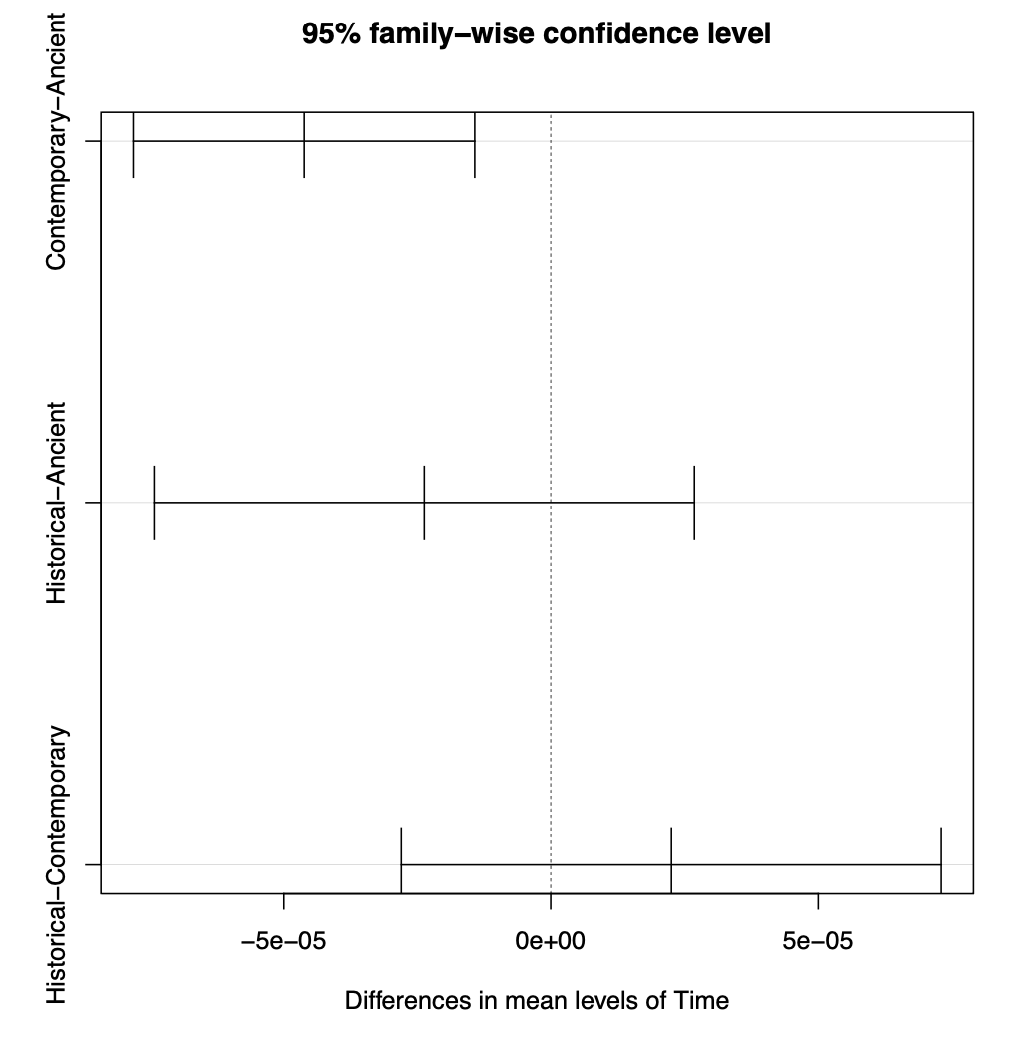


**Figure S15 – Tukey's HSD post-hoc test of the one-way ANOVA indicates significant differences in heterozygosity between the ancient and contemporary samples.** Each bar represents the 95% confidence window of significance in differences between mean heterozygosity over time. Bars that overlap with 0 suggest there is no significant difference, where bars that do not overlap with 0 suggest a significant difference. Here, the ancient samples are significantly different from the contemporary samples, with a mean difference of –4.62e-5 between contemporary and ancient heterozygosity (where contemporary is lower than ancient) and p=0.002. The difference between historical and ancient is –2.37 (where historical is lower; p=0.51) and between historical and contemporary is 2.25 (where historical is higher; p=0.55).

**Works Cited**

Alheit, J., & Hagen, E. (1997). Long-term climate forcing of European herring and sardine populations. *Fisheries Oceanography*, *6*(2), 130–139. https://doi.org/10.1046/j.1365-2419.1997.00035.x

Atmore, L. M., Martínez-García, L., Makowiecki, D., André, C., Lõugas, L., Barrett, J. H., & Star, B. (2022). Population dynamics of Baltic herring since the Viking Age revealed by ancient DNA and genomics. *Proceedings of the National Academy of Sciences*, *119*(45), e2208703119. https://doi.org/10.1073/pnas.2208703119

Barrett, J. H. (2016). Medieval Sea Fishing, AD 500-1550: Chronology, Causes and Consequences. In J. H. Barrett & D. C. Orton (Eds.), *Cod & Herring: The Archaeology & History of Medieval Sea Fishing* (pp. 250–272). Oxbow Books.

Barrett, J. H., Locker, A. M., & Roberts, C. M. (2004). The origins of intensive marine fishing in medieval Europe: The English evidence. *Proceedings of the Royal Society of London. Series B: Biological Sciences*, *271*(1556), 2417–2421. https://doi.org/10.1098/rspb.2004.2885

Bond, J. M., & O’Connor, T. P. (1999). Bones from medieval deposits at 16-22 Coppergate and other sites in York. The archaeology of York. The animal bones 15/5. *YAT & CBA*.

Corten, A. (1999). The reappearance of spawning Atlantic herring (Clupea harengus) on Aberdeen Bank (North Sea) in 1983 and its relationship to environmental conditions. *Canadian Journal of Fisheries and Aquatic Sciences*, *56*, 2051–2061. https://doi.org/10.1139/f99-142

Daan, N., Bromley, P. J., Hislop, J. R. G., & Nielsen, N. A. (1990). Ecology of North Sea Fish. *Netherlands Journal of Sea Research*, *26*(2–4), 3430486.

Galloway, J. (2017). Fishing in medieval England. In *The Sea in History: The Medieval World / La Mer dans L’Historie: Le Moyen Age* (pp. 629–642). Océanides Association (Boydell Press).

Hall, R. (1994). *Viking Age York*. Batsford Ltd.

Harland, J. F., Jones, A. K. G., Orton, D. C., & Barrett, J. H. (2016). Fishing and fish trade in medieval York: The zooarchaeological evidence. In J. H. Barrett & D. C. Orton (Eds.), *Cod and Herring: The Archaeology and History of Medieval Sea Fishing* (pp. 172–204). Oxbow Books.

Höglund, H. (1972). *On the Bohuslän herring during the great herring fishery period in the eighteenth century*.

Horne, T. (2021). *A Viking Market Kingdom in Ireland and Britain: Trade Networks and the Importation of a Southern Scandinavian Silver Bullion Economy*. Routledge. https://doi.org/10.4324/9780429341625

Jahnke, C. (2000). *Das Silber des Meeres: Fang und Vertrieb von Ostseehering zwischen Norwegen und Italien (12.-16. Jahrhundert): Vol. Bd. 49* (Quellen und Darstellungen zur hansischen Geschichte. Neue Folge). Böhlau.

Keaveney, E. (2005). *Fish Trade in York: Bones from Blue Bridge Lane and Fishergate House*. Unpublished MSc Dissertation, University of York.

Kowaleski, M. (2003). The Commercialization of the Sea Fisheries in Medieval England and Wales. *International Journal of Maritime History*, *15*(2), 177–231. https://doi.org/10.1177/084387140301500212

Kowaleski, M. (2016). The Early Documentary Evidence for the Commercialisation of the Sea Fisheries in Medieval Britain. In J. H. Barrett & D. C. Orton (Eds.), *Cod & Herring: The Archaeology & History of Medieval Sea Fishing* (pp. 23–41). Oxbow Books.

Laarman, F., & Lauwerier, R. (1996). *1996c: Laarman & Lauwerier: Vlees vis en gevogelte [in Dutch; Meat, fish and fowl; finds (1425-1500) from a cesspit of the House “Ín den Struys”, at Veere, the Netherlands ]* (pp. 91–99).

Novo, I., Ordás, P., Moraga, N., Santiago, E., Quesada, H., & Caballero, A. (2023). Impact of population structure in the estimation of recent historical effective population size by the software GONE. *Genetics Selection Evolution*, *55*(1), 86. https://doi.org/10.1186/s12711-023-00859-2

Poulsen, B. (2008). *Dutch Herring: An Environmental History, C. 1600-1860*. Amsterdam University Press.

Poulsen, B. (2010). The variability of fisheries and fish populations prior to industrialized fishing: An appraisal of the historical evidence. *Journal of Marine Systems*, *79*(3), 327–332. https://doi.org/10.1016/j.jmarsys.2008.12.011

Serjeantson, D., & Woolgar, C. M. (2006). Fish Consumption in Medieval England. In C. M. Woolgar, D. Serjeantson, & T. Waldron (Eds.), *Food In Medieval England: Diet and Nutrition* (p. 0). Oxford University Press. https://doi.org/10.1093/oso/9780199273492.003.0008

Sicking, L., & Abreu-Ferreira, D. (2009). *Beyond the Catch: Fisheries of the North Atlantic, the North Sea and the Baltic, 900-1850*. BRILL.

Skre, D. (2007). *Kaupang in Skiringssal*.
